# A Fixed-Ratio Hybrid ARO-ALO Algorithm for Multi-Level Thresholding of Histopathological Colon Cancer Images

**DOI:** 10.3390/cancers18162656

**Published:** 2026-08-17

**Authors:** Muhammed Faruk Şahin, Can Eyüpoğlu, Oktay Karakuş

**Affiliations:** 1 Department of Computer Engineering, Istanbul Atlas University, 34408 Istanbul, Türkiye; muhammed.sahin@atlas.edu.tr; 2 Department of Computer Engineering, Atatürk Strategic Studies and Graduate Institute, National Defence University, 34330 Istanbul, Türkiye; 3 School of Computer Science and Informatics, Cardiff University, Cardiff CF24 4AG, UK; eyupogluc@cardiff.ac.uk; 4 Department of Computer Engineering, Turkish Air Force Academy, National Defence University, 34149 Istanbul, Türkiye

**Keywords:** multi-level thresholding, hybrid metaheuristics, clinical decision support systems, colon cancer, medical image segmentation

## Abstract

**Background/Objectives:** Accurate segmentation of histopathological images while preserving cellular morphology in computer-aided diagnostic systems is critically important for the diagnosis and staging of colon cancer. However, conventional metaheuristic algorithms performing multi-level thresholding on such complex tissues often suffer from premature convergence by becoming trapped in local optima as the search space increases. To address this limitation, this study proposes a new label-independent hybrid optimization algorithm focused on colon adenocarcinoma segmentation. **Methods:** The proposed algorithm hybridizes the global exploration capability of the Artificial Rabbit Optimization (ARO) algorithm with the local exploitation ability of the Ant Lion Optimization (ALO) algorithm through an optimized fixed transition ratio, thereby enabling efficient localization of cellular density valleys. **Results:** The principal findings obtained from the LC25000 colon cancer dataset demonstrate that the ARO-ALO algorithm achieves stable performance with high SSIM (0.8043) and FSIM (0.8595) scores while preserving the histopathological hierarchy. Furthermore, the preservation of diagnostic morphology after segmentation is statistically validated by the high Pearson (0.9870) and Spearman (0.9948) correlation coefficients. In addition, supplementary generalization experiments are conducted on the Oral Squamous Cell Carcinoma (OSCC) and pulmonary circulation vessels datasets to verify the tissue-agnostic nature of the algorithm. **Conclusions:** Consequently, the ARO-ALO algorithm emerges as an efficient alternative for clinical decision support systems.

## Simple Summary

The precise segmentation of histopathological images, coupled with the preservation of cellular morphology, is of critical importance for the diagnosis and staging of colon cancer. However, conventional algorithms employed in the analysis of complex tissue architectures frequently suffer from premature convergence, becoming trapped in local optima as the dimensionality of the search space increases. To mitigate these limitations, a novel, label-independent hybrid algorithm characterized by highly efficient optimization stability is proposed. The primary objective of this study is to delineate cellular density boundaries by systematically integrating global exploration capabilities across broad search spaces with localized exploitation capacities. The derived findings demonstrate that the proposed methodology yields high structural accuracy without compromising the intrinsic tissue hierarchy. The outcomes of this research are anticipated to provide a robust diagnostic infrastructure for clinical decision support systems, free from topological deformations, thereby circumventing the reliance on extensively annotated datasets.

## 1. Introduction

Colon cancer is one of the most prevalent and lethal types of cancer worldwide, where early diagnosis and accurate classification play a critical role in the treatment of the disease [1,2]. In colon cancer treatment, accurate detection and classification of tumors are primarily achieved through segmentation techniques applied in medical imaging [3,4]. However, these segmentation processes are challenging due to the need to separate tumors from healthy tissues and correctly classify regions with similar tissue characteristics. These challenges become even more complex due to factors such as heterogeneous tissue structures, low-contrast areas, and regional variations [5,6,7,8]. To overcome these challenges, multi-level thresholding techniques have become increasingly popular in recent years [9,10]. This technique stands out as a powerful method to enhance segmentation quality by effectively distinguishing different intensity levels in the image [11]. However, multi-level thresholding applications involve significant computational burdens and complexities in high-resolution and structurally complex images, as accurate threshold determination is required [12]. For this reason, multi-level thresholding processes are often challenging when using traditional methods. In this context, metaheuristic (MH) algorithms offer an important solution to address these challenges [13,14,15].

MH algorithms are nature-inspired heuristic search strategies capable of generating near-optimal solutions in high-dimensional search spaces [16,17]. These algorithms provide effective and fast solutions to problems where traditional methods fall short, and are particularly useful in medical image segmentation, where they are employed to determine suitable threshold values for distinguishing different tissue classes. In addition to improving segmentation accuracy, MH algorithms contribute to classification, volume measurement, structural analysis, and various advanced medical analyses [18,19]. In the literature, especially in studies involving computed tomography (CT)-based colon cancer images, multi-level thresholding methods based on MH algorithms have shown effective results [20,21]. However, in the diagnosis of colorectal cancer, histopathological images provide detailed morphological information at the cellular level, thereby enabling the differentiation of pathological subtypes [22,23,24,25]. However, the high resolution, color diversity, and tissue complexity of histopathological images make the segmentation process more challenging [26,27]. Therefore, the combination of multi-level thresholding with metaheuristic algorithms aims to not only enhance diagnostic accuracy but also make a significant contribution to the segmentation literature of histopathological images. These methods provide direct contributions, not only for research purposes but also for clinical applications. Accurate and rapid segmentation of histopathological images facilitates pathologists in reliably separating tumor cells, identifying subtypes, and making earlier decisions in treatment planning. The clinical integration of the proposed model reduces observer dependency and ensures more objective and reproducible results.

### Motivation and Contribution

Colon cancer is among the oncological diseases with the highest morbidity and mortality rates worldwide, and early diagnosis together with accurate staging plays a critical role in the success of treatment protocols. In digital pathology, the detection and classification of tumorous tissues through histopathological imaging techniques constitute a fundamental requirement. However, histopathological sections present substantial analytical challenges for image segmentation due to heterogeneous cellular structures, complex epithelium–stroma boundaries, staining variability, and low-contrast lumen regions [28,29,30]. Within the existing literature on computer-aided diagnostic systems, two major methodological barriers are particularly prominent. The first barrier is the strong dependence of recently popularized fully supervised deep learning models on large-scale labeled datasets. Owing to the discrete labeling logic embedded in training masks, these models tend to lose the natural intensity gradients of histopathological cells and exhibit overfitting on limited datasets, thereby generating high structural variance. The second barrier concerns the conventional standalone metaheuristic optimization algorithms employed to address this issue, which frequently suffer from premature convergence by becoming trapped in local optima as the number of multi-level thresholding parameters increases within the search space. In light of these observations, the need for next-generation hybrid systems in histopathological image segmentation has become evident—systems that do not require labeled data, preserve tissue intensity hierarchies, and exhibit high optimization stability. The primary motivation of this study is to propose a novel fixed-ratio hybrid algorithm (ARO-ALO) that mathematically combines the exploration capability of the Artificial Rabbit Optimization (ARO) algorithm in broad search spaces with the exploitation capacity of the Ant Lion Optimization (ALO) algorithm for intensifying local valley searches. While the existing literature predominantly focuses on radiological images such as CT or magnetic resonance imaging (MRI), hybrid metaheuristic designs specifically developed to resolve the microscopic tissue complexity of histopathological data remain extremely limited. The proposed method aims to eliminate the structural distortions introduced by labeling and training procedures through the direct analysis of cellular intensity distributions. Although fully supervised semantic segmentation models, such as U-Net and its variants, classify pixels by directly assigning them to categorical segmentation masks, this process inevitably disrupts the continuity of intrinsic cellular intensity gradients and increases the susceptibility of these models to domain-shift effects when applied across heterogeneous datasets. Within this context, the proposed ARO-ALO algorithm is not intended to serve as a competing architecture that replaces deep learning-based segmentation models. Rather, it is designed as a label-independent, morphologically faithful (high-fidelity) preprocessing and feature-extraction framework that preserves the original tissue topology, thereby enabling subsequent diagnostic analyses to be performed on structurally undistorted histopathological representations. Consequently, the proposed framework provides a transparent and robust diagnostic foundation for Clinical Decision Support Systems (CDSSs) by eliminating the risk of topological deformations introduced by supervised segmentation networks while maintaining the morphological integrity of the underlying tissue structures. In this context, the original contributions of the study to the literature are summarized as follows:

- A novel hybrid ARO-ALO algorithm is introduced, in which the transition between exploration and exploitation phases is governed by a fixed-ratio (λ). Based on comprehensive ablation studies, it was demonstrated that when the threshold value was set to 10 or higher, the proposed fixed-ratio transition mechanism was more effective in preventing the algorithm from becoming trapped in local minima.
- The developed hybrid algorithm is evaluated not only against conventional metaheuristics but also against contemporary state-of-the-art (SOTA) segmentation models recognized as benchmark methods in the literature, including Reptile Search Algorithm-Salp Swarm Algorithm (RSA-SSA) [13] and WDRIME [31]. Its superiority in structural preservation (SSIM/FSIM) at clinically meaningful threshold levels is statistically validated through correlation analyses.
- The optimization capability of the algorithm is assessed not only on the target colon adenocarcinoma dataset but also on Oral Squamous Cell Carcinoma (OSCC) and Pulmonary Circulation Vessels datasets, which possess entirely different morphological characteristics. These cross-dataset analyses confirm the generalizability of the ARO-ALO algorithm across diverse histopathological tissue types.
- It is objectively demonstrated that the proposed unsupervised algorithm preserves cellular structural integrity (SSIM) and diagnostic characteristics (FSIM) with significantly greater stability compared to CUNet3+, a contemporary label-dependent deep learning architecture. Furthermore, the model’s ability to accurately localize histopathological histogram valleys is quantitatively and qualitatively validated.

Accordingly, the remaining sections of this study are organized in a systematic flow. Section 2 reviews existing deep learning and meta-heuristic-based segmentation approaches in the literature and identifies gaps that require further research. Section 3 details the mathematical foundations and design rationale of the proposed hybrid ARO-ALO methodology. In Section 4, the performance of the proposed method is presented in comparison with a baseline dataset, external validation sets, ablation studies, and deep learning models. Finally, in Section 5 and Section 6, the clinical significance of the findings is discussed, and perspectives for future studies are presented.

## 2. Related Works

This section provides a comprehensive review of the existing literature on image thresholding techniques, categorizing relevant methods under their respective subheadings. This structure highlights the current state of the problem domain addressed in the study and outlines the fundamental characteristics of the methods used. The corresponding summary table of the relevant literature is presented in Table 1.

### 2.1. Deep Learning-Based Colon Segmentation Approaches

Colon segmentation is a critical step in colon cancer diagnosis and treatment planning. However, obtaining accurate and reliable segmentation with limited labeled data remains a significant challenge. To address this issue, a three-stage deep neural network system for automated colorectal cancer detection using CT scan images is proposed [32]. The proposed method uses the SegNet network for tumor localization and the InceptionResNet V2 network to classify the tumors as benign or malignant. In real-life CT scans from 37 patients, the proposed model achieves 95.8% ROI segmentation accuracy, a Dice coefficient of 0.6214, an IoU score of 69.75%, and a tumor classification accuracy of 95.83%. Thus, the proposed system, by leveraging automation, deep learning, and innovative parameter analysis, presents a reliable and viable alternative to traditional cancer diagnosis techniques. However, due to issues with computational efficiency, prediction accuracy, and data dependence, a new deep learning approach is proposed to improve colon segmentation accuracy even with limited data labeling [33]. The proposed method integrates 3D contextual information by using guided sequential episodic training. A query CT slice is segmented by leveraging a previously labeled CT slice (i.e., a supporting slice). The segmentation process begins with rectum detection using a Markov Random Field (MRF)-based algorithm, and Contrastive Learning is employed to enhance the discriminative power of features, thus improving segmentation accuracy. In experiments conducted on 98 patients, a Dice coefficient of 97.3% and a polyp preservation accuracy of 98.28% are achieved. Statistical analyses of the method show results within a 95% confidence interval, demonstrating robustness and reliability. Thus, the method plays a critical role in preserving the details of polyps for accurate automatic diagnosis. Additionally, when trained with limited labeled data, the model achieves a Dice coefficient of 97.15%, indicating that the proposed approach is effective even with smaller labeled datasets. However, the challenging nature of fine-detail segmentation and the limitation in distinguishing small structural differences remain drawbacks. Therefore, research in digital pathology is growing in order to develop more precise segmentation methods.

Digital pathology plays a critical role in tumor diagnosis and prognosis, while the high resolution and large size of Whole Slide Image (WSI) images particularly limit the effectiveness of current segmentation methods. To address this issue, deep learning (DL) methods for tissue classification and segmentation in histopathological images are proposed [34]. The proposed method focuses on classifying colon cancer regions using Convolutional Neural Network (CNN) models such as AlexNet, VGG, ResNet, DenseNet, and Inception. Additionally, to compensate for the lack of richly labeled datasets, transfer learning techniques are employed to enhance performance. This strategy allows networks trained on large visual datasets to generate rich learned features. Experimental results show that the ResNet model tested on the AiCOLO colon cancer dataset achieves efficient classification results with an accuracy of up to 96.98%. Furthermore, pixel-based segmentation strategies using UNet and SegNet models are developed. These models are supported by multi-stage training strategies to overcome sparse annotations in histopathological images. With data augmentation and transfer learning, the UNet and SegNet models achieve accuracy rates of 76.18% and 81.22%, respectively. However, the SegNet model performs more efficiently with accuracy rates of 98.66%, 99.12%, and 78.39% on the CRC-5000, nct-crc-he-100k, and Warwick datasets, respectively. Nevertheless, the combination of large-scale images with large datasets leads to high computational costs. To overcome this limitation, automatic pattern recognition with deep learning techniques is becoming increasingly important. However, general preprocessing methods for high-resolution images carry the risk of losing crucial data such as high-frequency information and regions of interest. Therefore, a principal component analysis (PCA) and discrete wavelet transform (DWT)-based compressed domain segmentation approach is proposed [35]. In the proposed method, after analyzing each tile with neural networks, all predicted images are reconstructed using inverse discrete wavelet transform (IDWT)-based wavelet-weighted summation (WWE). This enhances the segmentation process by improving computational efficiency while preserving important image details. Training and validation are performed on 351 colorectal biopsy samples confirmed pathologically by two pathologists, and in analyses conducted on 39 test datasets, the average Dice score is 2.7%, pixel accuracy is 0.9%, and Jaccard score is 2.7%. Similarly, for histopathological image segmentation in WSI images, the Alter-AttUNet method is proposed [36]. The proposed method adds spatial attention gates to the Att-UNet model, aiming for more efficient segmentation. The proposed method achieves accuracy rates of 99.65%, 99.73%, and 79.03% on the NCT-CRC-HE-100K, CRC-5000, and Warwick datasets, respectively. However, the segmentation accuracy is limited by the small number of labeled samples and datasets filled with artifacts. In another study, a deep learning-based nuclear segmentation method using the U-Net biomedical image segmentation model is proposed for colon histology images [37]. The proposed method performs multi-class semantic segmentation, classifying each pixel in colon histology images as one of six different nucleus types or the background. Experimental image analysis on the Colon Nuclei Identification and Counting (CoNIC) Challenge dataset reveals a DSC value of 57.08% and an IoU value of 48.57% from 4981 histology images. Additionally, pixel-based classification analysis shows an accuracy of 69.61%, precision of 94.23%, and sensitivity of 94.48%. However, there is a need to improve accuracy statistics and achieve more efficient segmentation. Another study highlights the critical role of detecting gland morphology in colon histology images for determining the degree of colon cancer. However, manual segmentation is challenging, necessitating automatic segmentation methods. To address this issue, a diffusion model-based automatic gland segmentation method is proposed [38]. In the proposed method, the initial segmentation process for colon histology images is modeled as a noise reduction process based on the diffusion model. Additionally, to recover details lost during noise reduction, the method uses Instance Aware Filters and multi-scale Mask Branches to generate global masks instead of predicting local masks. Finally, Conditional Encoding is applied to strengthen the intermediate features with original image encoding, improving the difference between the object and the background. The proposed method is tested on the GlaS, CRAG, and RINGS datasets, with results showing F1 scores of 0.853 ± 0.054 and Dice scores of 0.906 ± 0.043 for CRAG, F1 scores of 0.941 ± 0.039 and Dice scores of 0.939 ± 0.060 for GlaS Test A, and precision of 0.893 ± 0.096 and Dice scores of 0.904 ± 0.091 for RINGS. These results demonstrate the improvement in segmentation accuracy provided by the proposed method.

### 2.2. Colon Cancer Segmentation Using Metaheuristic Algorithms

Colorectal cancer (CRC) plays a significant role in computer-aided diagnosis (CAD) systems for automatic polyp detection. Traditional machine learning algorithms have limited generalization capabilities and sensitivities, which has led deep learning algorithms to stand out in this field. Deep learning, particularly in colonoscopy images, has achieved successful results in early and accurate polyp detection. Therefore, the Artificial Bee Colony (ABC) algorithm is proposed to optimize the hyperparameters of YOLO algorithms [39]. The proposed method can easily integrate with all YOLO algorithms such as YOLOv3, YOLOv4, Scaled-YOLOv4, YOLOv5, YOLOR, and YOLOv7. The performance of Scaled-YOLOv4 shows over a 3% increase in mean average precision (mAP) and a 2% improvement in F1 score. Additionally, evaluations on the SUN and PICCOLO datasets show that this method improves the optimization of YOLO-based algorithms. However, the study has limitations due to insufficient dataset size, as the limited number of images and patients in public datasets highlights the need for more data to achieve high performance with deep learning algorithms. In this context, the accurate segmentation of colon cancer in medical images remains a challenging task due to the complexity of its morphology and the limited availability of annotated data. To improve this issue, a combination of a Hyperparameter-Tuning Attention U-Net and Pix2Pix Generative Adversarial Network (Pix2Pix-GAN) guided by the Sine Cosine Algorithm (SCA) is proposed for colon cancer segmentation and image synthesis [40]. In the proposed method, SCA optimizes the balance between the generator and discriminator dynamics, providing better convergence and stability. As a result, the method achieves an average Dice score of 0.9514, Intersection over Union (IoU) of 0.9123, F-beta score of 0.9636, and similarity index of 0.9430. Additionally, the Mean Absolute Error (MAE) is minimized to 0.01583. However, the computational complexity due to the use of SCA and numerous hyperparameters leads to high computational costs. Moreover, sensitivity to initial values results in performance variability depending on the initial hyperparameter configuration, which can hinder reaching global optima or effective exploration of the search space. To address this issue, a method using CNNs and the Fishier Mantis Optimizer (FMO) is proposed [41]. In this method, the FMO fine-tunes the CNN parameters to enhance convergence speed and performance. Experimental results show sensitivities, specificities, accuracies, and F1 scores of 94.87%, 96.19%, 97.65%, and 96.76%, respectively. However, the model’s generalization ability and accuracy in real-world environments remain limited due to the insufficient representation of rare pathologies in datasets, computational intensity, and limited validation studies. In another study, accurate segmentation of lesion regions in colonoscopy histopathology images is problematic, particularly due to changes in image quality following grayscale conversion and texture processing steps. To address this issue, an optimization method using Thresholding, Particle Swarm Optimization (PSO), Darwinian PSO (DPSO), and Fractional Order DPSO (FODPSO) algorithms is proposed [42]. The FODPSO algorithm achieves accuracy of 89%, Dice score of 0.68, and Jaccard score of 0.52 on the colonoscopy dataset, performing more efficiently than the artificial bee colony and firefly algorithms. However, converting colored histopathological colon cancer images to grayscale for thresholding causes a loss of color information, which negatively impacts segmentation accuracy and limits the method’s effectiveness. A review of the existing literature reveals that individual meta-intuitive algorithms face stability issues, particularly in the face of color variability and tissue heterogeneity in histopathological images, and highlights the high data dependency of deep learning models. These limitations form the fundamental basis and methodological necessity for the hybrid ARO-ALO algorithm proposed in this study, which manages the exploration-exploitation balance through an fixed-ratio mechanism. A comprehensive review of the existing literature reveals that deep learning- and metaheuristic-based approaches for histopathological image segmentation exhibit distinct structural advantages as well as critical limitations. Supervised deep learning models, including CNNs, U-Net, and their variants, have established a prominent position in the literature by achieving high semantic segmentation accuracy and efficient inference performance when trained on large-scale annotated datasets. However, their strict dependence on extensively pixel-wise labeled data substantially limits their practical applicability in medical image analysis, where obtaining high-quality annotations is both labor-intensive and costly. Furthermore, compelling these architectures to learn the discrete labeling paradigm imposed by externally generated ground-truth masks often results in the loss of the intrinsic intensity gradients naturally present in histopathological tissues, while simultaneously increasing the susceptibility of the models to overfitting under the illumination and staining conditions represented in the training data. In contrast, conventional metaheuristic algorithms offer a training-free optimization framework that enables adaptive thresholding directly on pixel intensity distributions without requiring annotated datasets. Nevertheless, existing approaches based on PSO, Grey Wolf Optimizer (GWO), and conventional hybrid metaheuristics remain insufficient for handling the multimodal and asymmetric cellular intensity distributions inherent in histopathological images. This limitation becomes particularly evident in multilevel thresholding scenarios (T≥8), where the combinatorial expansion of the search space significantly increases optimization complexity, causing these algorithms to become trapped in shallow local optima and suffer from premature convergence. Moreover, the majority of existing hybrid methods regulate the balance between exploration and exploitation using static control parameters, resulting in inefficient utilization of the optimization budget in complex tissue structures characterized by intertwined epithelial and stromal regions. Consequently, a significant research gap exists in the current literature for a new generation of hybrid optimization architectures that can simultaneously overcome the limitations of both paradigms by eliminating the need for annotated datasets and computationally intensive training procedures, preserving the morphological topology of histopathological tissues, and maintaining robust search stability under high-complexity optimization problems through fixed-ratio mechanisms. The proposed ARO-ALO architecture is designed to address these fundamental shortcomings by introducing a fixed-ratio transition mechanism that adaptively regulates the exploration–exploitation balance throughout the optimization process.

**Table 1:** Summary of colorectal cancer segmentation studies reported in the literature.

Ref.	Method	Dataset	Metrics
[32]	iScan	BALCO Medical Center *	ROI segmentation accuracy (95.8%), dice coefficient (0.6214), IoU (69.75%), tumor classification accuracy (95.83%)
[33]	Markov Random Field-Based and Contrastive Learning-Supported Sequential Episodic Training Framework (G-SET-DCL)	Synapse Dataset [43]	Dice coefficient (97.3%), polyp information preservation accuracy (98.28%)
[34]	Improved UNet and SegNet	AiCOLO, CRC-5000, NCT-CRC-HE-100K, Warwick [34]	Accuracy rate (ResNet: 96.98%, SegNet: up to 99.12%
[35]	PCA- and Inverse Discrete Wavelet Transform (IDWT)-Based Wavelet-Weighted Ensemble (WWE)-Supported Compressed Domain Segmentation	Yeouido St. Mary’s Hospital, H&E stained-WSIs of colorectal biopsy specimens [35]	Dice score (2.7%), pixel accuracy (0.9%), Jaccard score (2.7%)
[36]	Alter-AttUNet model	AiCOLO, NCT-CRC-HE-100K, CRC-5000, Warwick [34]	Accuracy: (NCT-CRC-HE-100K: %99.65, CRC-5000: 99.73%, Warwick: 79.03%)
[37]	U-Net-based	Colon Nuclei Identification and Counting (CoNIC) Challenge [44]	DSC (57.08%), IoU (48.57%), Piksel accuracy (69.61%), precision (94.23%), recall (94.48%)
[38]	Diffusion Model-Based	GlaS [45], CRAG	F1 score (0.853–0.941), Dice score (0.904–0.939), Precision (0.893)
[39]	Hyperparameter-Optimized YOLO Architectures Using the ABC Algorithm	SUN [46] and PICCOLO [47]	mAP improved (>3%), F1 score improved (2%)
[40]	SCA-Guided Hyperparameter Optimization of Attention U-Net and Pix2Pix-GAN	Kvasir-SEG [48]	Dice score (0.9514), IoU (0.9123), F-beta score (0.9636), similarity index (0.9430), MAE (0.01583)
[41]	CNN–Fishier Mantis Optimizer model	LC25000 [49]	sensitivity (94.87%), specificity (96.19%), accuracy (97.65%), F1 score (96.76%)
[42]	PSO, Darwinian PSO and FODPSO	Digestpath2019-grand challenge [50]	Accuracy (0.89), Dice score (0.68), Jaccard score (0.52)

*: Detailed participant information is not publicly available to protect patient privacy and comply with ethical requirements.

## 3. Methodology

**Figure 1:**
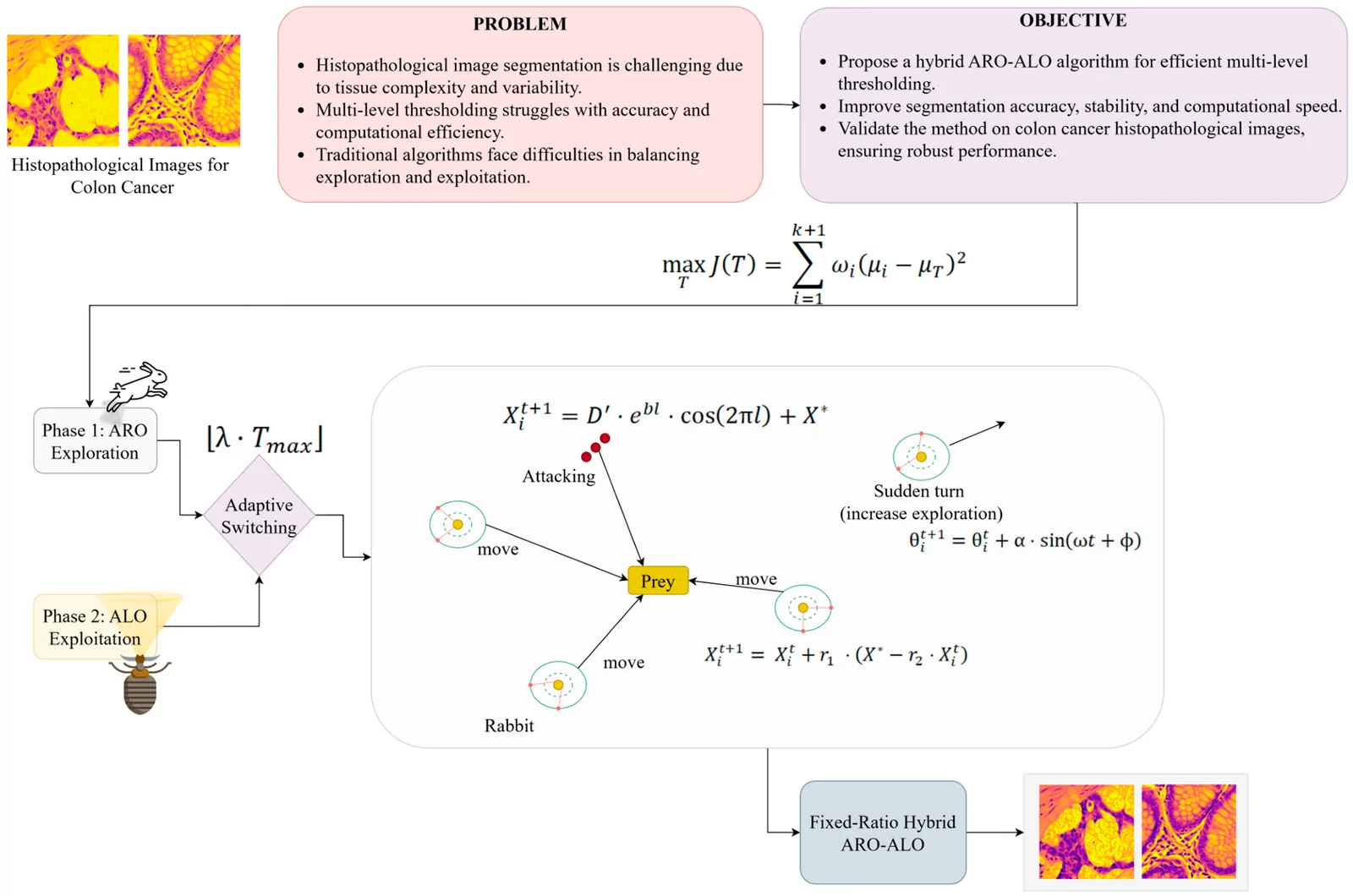
Graphical abstract of the proposed method. Background colors are utilized to conceptually delineate the problem statement, primary objectives, and distinct operational phases. Within the depicted optimization dynamics, agents converging toward the ‘Prey’ denote the global exploration mechanism driven by the Artificial Rabbit Optimization (ARO) algorithm, whereas the ‘Attacking’ elements signify the local exploitation phase executed by the Ant Lion Optimization (ALO) algorithm.

In this study, a novel metaheuristic algorithm is proposed for performing multi-level thresholding on colon cancer histopathological images, where the ARO and ALO algorithms are hybridized. The selection of ARO and ALO is based on their complementary strengths. ARO, with its high exploration capacity, ensures the maintenance of population diversity and efficiently scans complex search spaces. In contrast, ALO provides strong exploitation capabilities by refining solution candidates around promising regions through its spiral and encircling behaviors. The integration of these two algorithms offers a balanced search strategy suitable for the multi-level thresholding process of histopathological images, which requires both global exploration and local refinement. This section details the objective function of the proposed hybrid algorithm, the design of the hybrid algorithm, and its time complexity. The graphical abstract is presented in Figure 1.

### 3.1. Definition of the Objective Function

In this study, the proposed fixed-ratio hybrid algorithm for solving the multi-level thresholding problem presents an optimization framework aimed at maximizing the accuracy of image segmentation. The objective function used in this context is based on the Otsu [51] variance maximization principle. The goal is to minimize the total within-class variance while maximizing the between-class variance in the image. In accordance with this principle, the total variance among the classes, defined by the set of multiple threshold values $T=\{t_{1},t_{2},\ldots ,t_{k}\}$, is represented in Equation (1). Here, k denotes the dimensionality of the optimization search space for a given segmentation task and is consequently fixed according to the predefined value of k.

(1) $$\underset{T}{\max}J\left(T\right)=\sum_{i=1}^{k+1}\omega_{i}{\left(\mu_{i}-\mu_{T}\right)}^{2}$$

Here, $\omega_{i}$ represents the probability of the i−th class, $\mu_{i}$ is the mean of the i−th class, and $\mu_{T}$ is the global mean of the entire image. The probability $\omega_{i}$ and the mean $\mu_{i}$ of each class are given in Equation (2).

(2) $$\omega_{i}=\sum_{j=t_{i-1}+1}^{t_{i}}p_{j},\;\mu_{i}=\frac{1}{\omega_{i}}\sum_{j=t_{i-1}+1}^{t_{i}}j\cdot p_{j}$$

Here, $p_{j}$ represents the normalized histogram value of the j-th gray level. The overall mean is given in Equation (3).

(3) $$\mu_{T}=\sum_{j=0}^{L-1}j\cdot p_{j}$$

Thus, J(T) aims to find the optimal set of thresholds for multi-level thresholding. This function is optimized by the proposed hybrid algorithm. The threshold search space is defined as continuous real numbers in the range (0, 255). Therefore, unlike the classical Otsu method, threshold values can be precisely optimized without being restricted to fixed grid points. This allows for the achievement of the global optimum solution, especially in multi-level thresholding problems, where the fitness surface may be complex and contain multiple local peaks. The proposed approach offers more efficient segmentation accuracy and flexible analysis capabilities compared to deterministic methods.

### 3.2. Proposed ARO-ALO Methodology

The proposed fixed-ratio hybrid methodology is built using a fixed-ratio hybrid structure combining the ARO [52] and ALO [53]. This hybrid approach organizes the exploration and exploitation phases in a complementary manner to solve the optimization problem. The ARO algorithm operates during the exploration phase, while the ALO algorithm comes into play during the exploitation phase. This structure ensures that the algorithm exhibits flexible and balanced performance in both phases. The ARO algorithm is an optimization algorithm inspired by the exploration behavior of rabbits. In the ARO algorithm, a broad area of the solution space is explored to investigate potential solutions. Each individual (solution) updates its new position based on both its previous position and the best-found solution. This process allows the algorithm to spread across different regions of the solution space. The position of individuals in the ARO algorithm is updated according to Equation (4).

(4) $$X_{i}^{t+1}=X_{i}^{t}+r_{1}\;\cdot \left(X^{*}-r_{2}\cdot X_{i}^{t}\right)$$

Here, $X_{i}^{t}$ represents the position of the i-th individual at the t-th iteration, and $X^{*}$ denotes the global best solution found so far across all iterations from the 1st to the t-th iteration. The parameters $r_{1}$ and $r_{2}$ are independent random variables uniformly distributed in the interval $\left[0,1\right]$. Thus, each individual is guided towards the solution by considering both its past position and the position of the best solution. In this way, individuals explore a wide range of the solution space, investigating various possibilities. Alternatively, the positions of the individuals are updated based on the differences with the best solution, as expressed in Equation (5).

(5) $$X_{i}^{t+1}=X_{i}^{t}+r_{3}\cdot D$$

Here, $D=X^{*}-X_{i}^{t}$ represents the difference vector between the global best solution and the current individual’s position. The parameter $r_{3}$ is a random variable drawn from a continuous uniform distribution, denoted as $U\left(0,1\right)$. This strategy directs the individuals towards the best solution while maintaining randomness, thus preventing premature convergence. During the exploration phase, the direction of the individuals dynamically changes and, through random oscillations over time, encourages the exploration of more diverse solutions. This behavior is expressed in Equation (6).

(6) $$\theta_{i}^{t+1}=\theta_{i}^{t}+\alpha \cdot \sin\left(\omega t+\phi \right)$$

Here, $\theta_{i}^{t}$ denotes the directional angle of the i-th individual at the t-th iteration, which dictates its stochastic movement trajectory across the high-dimensional search space. Additionally, α represents the amplitude, ω the angular frequency, and ϕ the phase shift of the oscillation. This approach facilitates the exploration of a broader solution space during the exploration phase.

The ALO algorithm is an optimization algorithm inspired by the hunting behavior of ant lions. ALO focuses on searching within a narrower region of the solution space, concentrating on better solution areas. This phase starts with the best solution obtained during the exploration phase of ARO and performs an intensive local search around this solution. In the ALO algorithm, the position of each individual is updated in a circular motion around the best solution, as described in Equation (7).

(7) $$X_{i}^{t+1}=X^{*}-A\cdot \left|C\cdot X^{*}-X_{i}^{t}\right|$$

Here, *A* and *C* are the parameters of the algorithm, and *r* represents a uniformly distributed random number in the range (0, 1). This ensures that the search starts over a broader area, but gradually helps the solution converge within a narrower region as the process progresses. Additionally, ALO employs a spiral update strategy, where individuals move around the best solution. This spiraling motion is described by Equation (8).

(8) $$X_{i}^{t+1}=D^{\prime }\cdot e^{bl}\cdot \cos\left(2\pi l\right)+X^{*}$$

Here, $D^{\prime }$ represents the distance between $X^{*}$ and $X_{i}^{t}$, *b* is the spiral density, and l is a uniformly distributed random number in the range (−1, 1). The use of this spiral ensures that the individuals approach the best solution more intensively.

The proposed fixed-ratio hybrid methodology combines the strengths of the ARO and ALO algorithms. In this hybrid model, the first phase uses the ARO algorithm for a broad exploration of the solution space. The exploration phase ensures the investigation of various regions within the solution space, starting with ARO’s random-based rabbit movements across the space. In the second phase, ALO is employed. ALO begins with the best solution found in the exploration phase and conducts an intense local search around that solution. This phase ensures the solution is optimized more precisely. The transition between these two phases allows the algorithm to perform effectively in both stages. This transition is controlled by an transition rate (λ) that ensures a more efficient and balanced solution throughout the overall optimization process. The number of iterations for $T_{\mathrm{ARO}}$ and $T_{\mathrm{ALO}}$ is calculated based on the total iteration count ($T_{\max}$) and the rate λ, as shown in Equation (9).

(9) $$T_{\mathrm{ARO}}=\left\lfloor \lambda \cdot T_{\max}\right\rfloor ,\;T_{\mathrm{ALO}}=T_{\max}-T_{\mathrm{ARO}}$$

This transition structure allows the solution to explore a wide search space and then focus around the best solution. As a result, the proposed hybrid ARO-ALO algorithm possesses the capacity to both explore a wide solution space and exploit the best solution obtained, enabling better results in complex and multi-modal solution spaces. This approach leverages the strengths of both algorithms, providing a more efficient and flexible solution. The hybrid methodology ensures that ARO performs extensive exploration in the exploration phase, while ALO conducts more intense and deeper searches in the exploitation phase. In addition to these contributions, the fundamental rationale underlying the design of the hybrid architecture is based on the hierarchical nature of histopathological images. The ARO phase performs a coarse exploration of extensive tissue regions, such as stroma and lumen areas, to determine an initial threshold interval, whereas the ALO phase intensifies the search around these candidate regions through a spiral-based exploitation mechanism to precisely delineate cellular nuclear boundaries. This two-stage process enables the preservation of clinically significant morphological details through a robust numerical optimization framework.

### 3.3. Complexity Analysis and Pseudo-Code Representation of the Proposed Method

**Figure 2:**
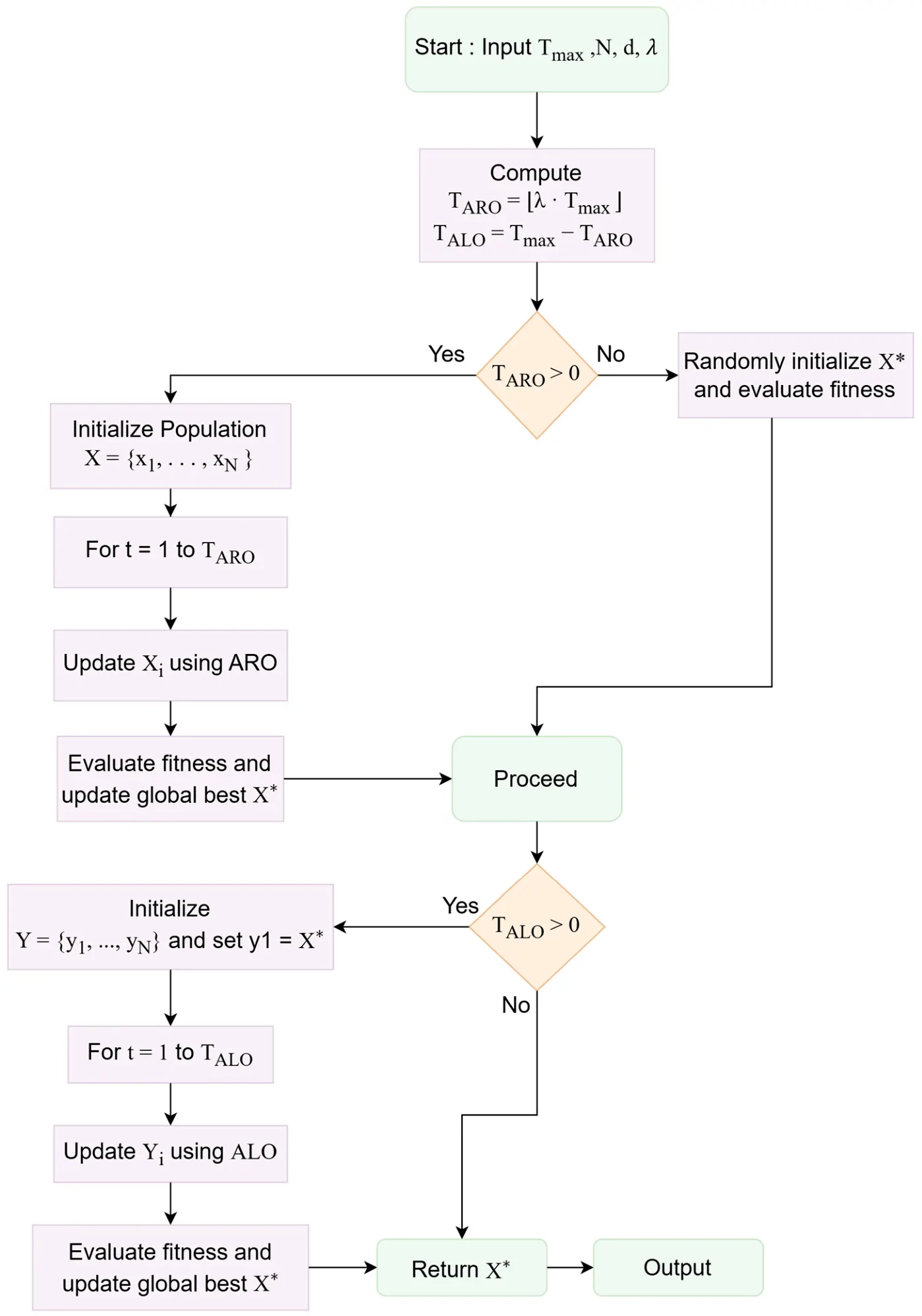
Flowchart of the proposed method.

In the proposed hybrid ARO-ALO algorithm, the position updates of agents with a population size N calculated at each iteration. In the specific context of the multi-level thresholding problem, N denotes the total number of search agents (candidate solution vectors) evaluating the histogram, while the problem dimension d directly corresponds to the pre-defined number of threshold levels being optimized. The position update operation is proportional to this problem size d, resulting in a computational cost of $O\left(d\right)$ per agent. Additionally, the fitness value of each individual is computed through an Otsu-based objective function. Since the Otsu function operates on the grayscale histogram, it incurs an additional computational burden of $O\left(L\right)$ at each evaluation, where L represents the number of gray levels in the image, typically L = 256. Therefore, the total computational load per iteration is expressed as $O\left(N\times \left(d+L\right)\right)$, which includes the computation for each of the N agents *d*-dimensional solution vectors and the grayscale histogram calculations for each individual. When the total number of iterations is T, the overall asymptotic computational load is initially expressed as $O\left(N\times T\times \left(d+L\right)\right)$. Given that the number of grayscale levels is a fixed constant (L=256), this additive term does not dictate the asymptotic growth rate of the algorithm. Therefore, in strict accordance with Big-O notation principles, the overall time complexity inherently simplifies to $O\left(N\times T\times d\right)$. Here, the phase transition mechanism does not significantly increase the overall complexity, as it only introduces a decision process with a constant cost of $O\left(1\right)$. This analysis shows that the proposed Hybrid ARO-ALO algorithm has an asymptotic complexity of a similar order to that of classical metaheuristic methods, and, thanks to its hybrid phase structure, it provides additional advantages in terms of solution quality and convergence speed. The general steps of the algorithm are outlined in the pseudo-code in Algorithm 1, and the flowchart is shown in Figure 2.
**Algorithm 1.** Fixed-Ratio Hybrid ARO-ALO Algorithm1: Input: Total iterations $T_{max}$, population size N, dimension d, switch ratio λ 2: Output: Optimal solution $X^{*}$ and its corresponding fitness value 3: Set $T_{aro}=\left\lfloor \lambda \cdot {\;T}_{max}\right\rfloor$ and $T_{alo}=T_{max}-T_{aro}$ 4: **if** $T_{aro}>0$
**then**                                                                    /*ARO Exploration Phase*/ 5:            Initialize population $X=\left\{X_{1},X_{2},\ldots ,X_{N}\right\}$ randomly within the search space. 6:            **for** t=1 to $T_{aro}$ **do** 7:                       **for** each individual $X_{i}\in X$
**do** 8:                       Update $X_{i}$ using ARO movement: $X_{i}^{t+1}=X_{i}^{t}+r_{1}\left(X^{*}-r_{2}X_{i}^{t}\right)$ 9:                       **end for** 10:                   Evaluate fitness $f\left(X_{i}\right)$ for all $X_{i}$ 11:                   Update global best $X^{*}\leftarrow \arg\max f\left(X_{i}\right)$ 12:          **end for** 13: **else** 14:          Randomly initialize best solution $X^{*}$ within bounds 15:          Evaluate fitness $f\left(X^{*}\right)$ 16: **end if** 17: **if** $T_{alo}>0$ **then**                                                                   /*ALO Exploitation Phase*/ 18:          Set $Y_{1}=X^{*}$                          /*Carry over the best solution from ARO phase*/ 19:          **for** t=1 to $T_{alo}$ **do** 20:                     **for** each $Y_{i}\in Y$ **do** 21:                                 Compute A=2a⋅r−a,C=2r, where $a=2-\frac{2t}{T_{alo}}$, $r∼U\left(0,1\right)$ 22:                                 **if** $\left|A\right|<1$ **then**                          /*Encircling prey (exploitation) */ 23:                                          **Encircling Prey:**
$Y_{i}^{t+1}=X^{*}-A\cdot \left|C\cdot X^{*}-Y_{i}^{t}\right|$ 24:                                 **else** 25:                                          **Spiral Movement:** $Y_{i}^{t+1}=D^{\prime }\cdot e^{bl}\cos\left(2\pi \cdot l\right)+X^{*}$ 26:                                 **end if** 27:                     **end for** 28:                     Evaluate fitness $f\left(Y_{i}\right)$ and update $X^{*}$ if a better solution is found 29:            **end for** 30: **end if** 31: Return $X^{*},f\left(X^{*}\right)$


## 4. Experiments and Results

In this section, the proposed ARO-ALO fixed-ratio hybrid algorithm is used to evaluate the segmentation performance of colon cancer in histopathological images. For comparison, the algorithm is compared with PSO, GWO, ARO, ALO, and the recently most efficient method in the literature, WDRIME [31]. In this study, the LC25000 dataset [49], which contains histopathological color images of colon cancer, is used. Originally, the LC25000 dataset includes 10,000 histopathological images of colon cancer, but this number is increased through augmentation techniques, including horizontal/vertical flipping (with a 50% probability) and ±25° rotation, to balance class distribution. Within the dataset, image clusters of benign colon tissue and colon adenocarcinomas are provided separately. Since metaheuristic thresholding methods do not undergo training on data, unlike deep learning models, the augmentation is only performed to expand the dataset and was not applied during optimization. For fair evaluation, 100 images are used for testing. All images are resized to 256 × 256 pixels to reduce computational load and ensure consistency in histogram-based thresholding, while the original (0, 255) 8-bit intensity range is strictly preserved to allow for accurate multi-level variance calculations.

### 4.1. Experiment Setup

In this study, the use of the range T=2–12 for multi-level thresholding problems is based on reports indicating that these boundaries provide sufficient separation for practical segmentation applications [54,55]. Lower numbers of thresholds (T<2) reduce the discriminative power of segmentation, while higher numbers of thresholds (T>12) neither produce meaningful subregions nor justify the increased computational cost. Therefore, the selected range of 2–12 is both consistent with the literature and represents realistic segmentation scenarios. For all these threshold values, the number of iterations is fixed at 100 to ensure a fair comparison among algorithms and control the computational costs. Each experiment is conducted with 10 independent runs to evaluate the variance due to the stochastic nature of the algorithms. The population size is set to 30 for all algorithms. Although it is acknowledged that the performance of metaheuristic (MH) algorithms is highly sensitive to parameter settings, all baseline algorithms are run with the commonly used default settings in the literature to ensure comparability. More extensive hyperparameter optimization or adaptive parameter control is left as a direction for future research. Detailed parameters and configurations of the relevant algorithms are presented in Table 2, and hardware and software specifications are provided in Table 3. For the GWO algorithm, the parameter a is gradually decreased from 2 to 0 according to a linear decay strategy, supported by research, thus ensuring a balance between exploration and exploitation. In PSO, the inertia weight w is in the range (0, 1), and the cognitive and social parameters $c_{1}$ and $c_{2}$ are in the range (1, 3). In the WDRIME model, the β range (0.2–0.8) is selected in alignment with fine-tuned adjustments to improve performance. The α parameter used in the ARO algorithm plays a crucial role in calculating the energy factor and defining the rabbit’s escape strategies. This parameter is set to 2 and maintains balance during the search process. In the ALO algorithm, the mechanisms that update the ant-lion traps are run with default parameters. Therefore, the adaptive random walk and elitist-based update strategies inherent in the algorithm do not require any additional parameter tuning. In the proposed hybrid ARO-ALO algorithm, one of the key parameters, the switch ratio λ, is determined to be 0.6 following sensitivity tests conducted within the range (0.4, 0.7). These tests show that λ=0.6 provides the most consistent and stable results across different threshold levels. This value means that 60% of the total iterations are spent on the exploration phase with ARO, while the remaining 40% are spent on the exploitation phase with ALO. As a result, the proposed hybrid structure combines ARO’s strong exploration capacity with ALO’s elitist-based exploitation power, while the careful selection of parameters maximizes both comparability and computational efficiency. All calculations are performed in a serial processing manner, allowing for a realistic comparison of the computational load across different algorithms.

### 4.2. Results

In this section, the performance of the proposed ARO-ALO algorithm is analyzed in detail through a comparative study with the commonly preferred PSO, GWO, ARO, ALO, and the hybrid adaptive WDRIME algorithms. In this context, Peak Signal-to-Noise Ratio (PSNR), Structural Similarity Index Measure (SSIM), and Feature Similarity Index Measure (FSIM) metrics are quantitatively reported, and statistical differences between the algorithms are evaluated. PSNR is considered a fundamental quality metric that indicates the closeness of the image obtained after multi-level thresholding to the original data and expresses the degree of information loss or noise effects numerically. Higher PSNR values signify better image quality and demonstrate the effectiveness of the optimization strategy. SSIM is another important quality metric that measures structural similarity and evaluates the structural integrity of the segmentation. SSIM values reflect how structurally similar the segmentation results are to the original image. FSIM, on the other hand, is a metric that considers phase and amplitude information in the image to measure image quality and is of great importance in terms of human visual system compatibility.

#### 4.2.1. Results for Colon Adenocarcinomas Class

**Figure 3:**
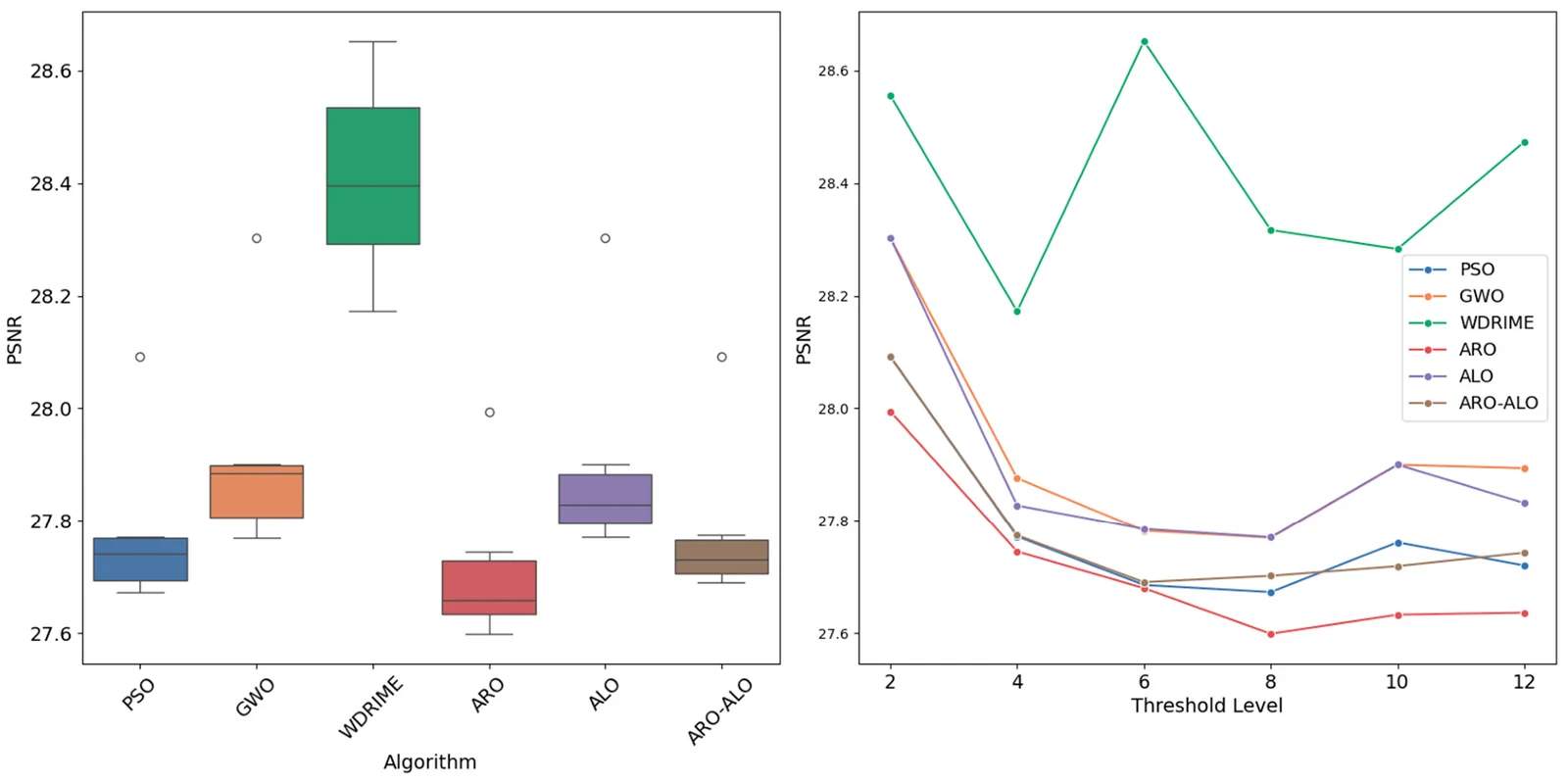
PSNR performance for colon adenocarcinomas images according to algorithms.

When examining the PSNR metric for Colon Adenocarcinoma images, the proposed ARO-ALO algorithm demonstrates robust performance across all threshold levels, achieving comparable or superior results relative to the other algorithms, as presented in Table 4 and Figure 3. In the subsequent comparative analyses, the box plots presented in the left panels of the figures illustrate the aggregated statistical distribution of the corresponding metric across all evaluated threshold levels ($T\in \left[2,12\right]$), whereas the line plots in the right panels display the mean performance trend at each specific threshold. At the T = 2 level, the ARO-ALO algorithm achieves a PSNR value of 28.0920 ± 0.4771 dB, producing similar results to the PSO (28.0920 ± 0.4771 dB) and GWO (28.3029 ± 0.4854 dB) algorithms. At this low threshold level, the PSNR values of all algorithms are quite close, with ARO-ALO’s stable performance being particularly noteworthy. At the T = 4 level, ARO-ALO achieves a PSNR of 27.7737 ± 0.2170 dB, similar to the PSO (27.7718 ± 0.2158 dB) and GWO (27.8760 ± 0.2430 dB) algorithms. However, the WDRIME (28.5558 ± 0.4805 dB), ARO (27.9947 ± 0.5031 dB), and ALO (28.3033 ± 0.4852 dB) algorithms show slightly more efficient PSNR values. This performance can be considered as ARO-ALO providing balanced performance with low standard deviation, demonstrating stability. At the T = 8 level, ARO-ALO produces a PSNR of 27.7018 ± 0.2021 dB, offering competitive performance compared to the PSO (27.6731 ± 0.1949 dB) and GWO (27.7695 ± 0.2163 dB) algorithms. At this level, ARO-ALO exhibits slight efficiency improvements and offers superior segmentation quality with its stability. Additionally, the WDRIME (28.3168 ± 0.2713 dB), ARO (27.5993 ± 0.1701 dB), and ALO (27.7703 ± 0.2430 dB) algorithms provide similar or higher PSNR values. At the T = 10 level, ARO-ALO achieves a PSNR of 27.7190 ± 0.2452 dB, which is slightly lower than the PSO (27.7612 ± 0.3122 dB) and GWO (27.9000 ± 0.3179 dB) algorithms. However, the ARO (27.6330 ± 0.1864 dB) and ALO (27.9003 ± 0.2374 dB) algorithms yield results more similar to WDRIME (28.2830 ± 0.5842 dB) and PSO. At higher threshold levels, ARO-ALO’s low standard deviation makes it a more stable option. At the T = 12 level, ARO-ALO achieves a PSNR of 27.7427 ± 0.2642 dB, yielding results close to the PSO (27.7201 ± 0.2449 dB) and GWO (27.8940 ± 0.2192 dB) algorithms. However, the WDRIME (28.4733 ± 0.4692 dB), ARO (27.6366 ± 0.2191 dB), and ALO (27.8319 ± 0.2220 dB) algorithms yield slightly more efficient PSNR values. The low standard deviation of ARO-ALO at this level demonstrates its ability to maintain stable performance.

**Figure 4:**
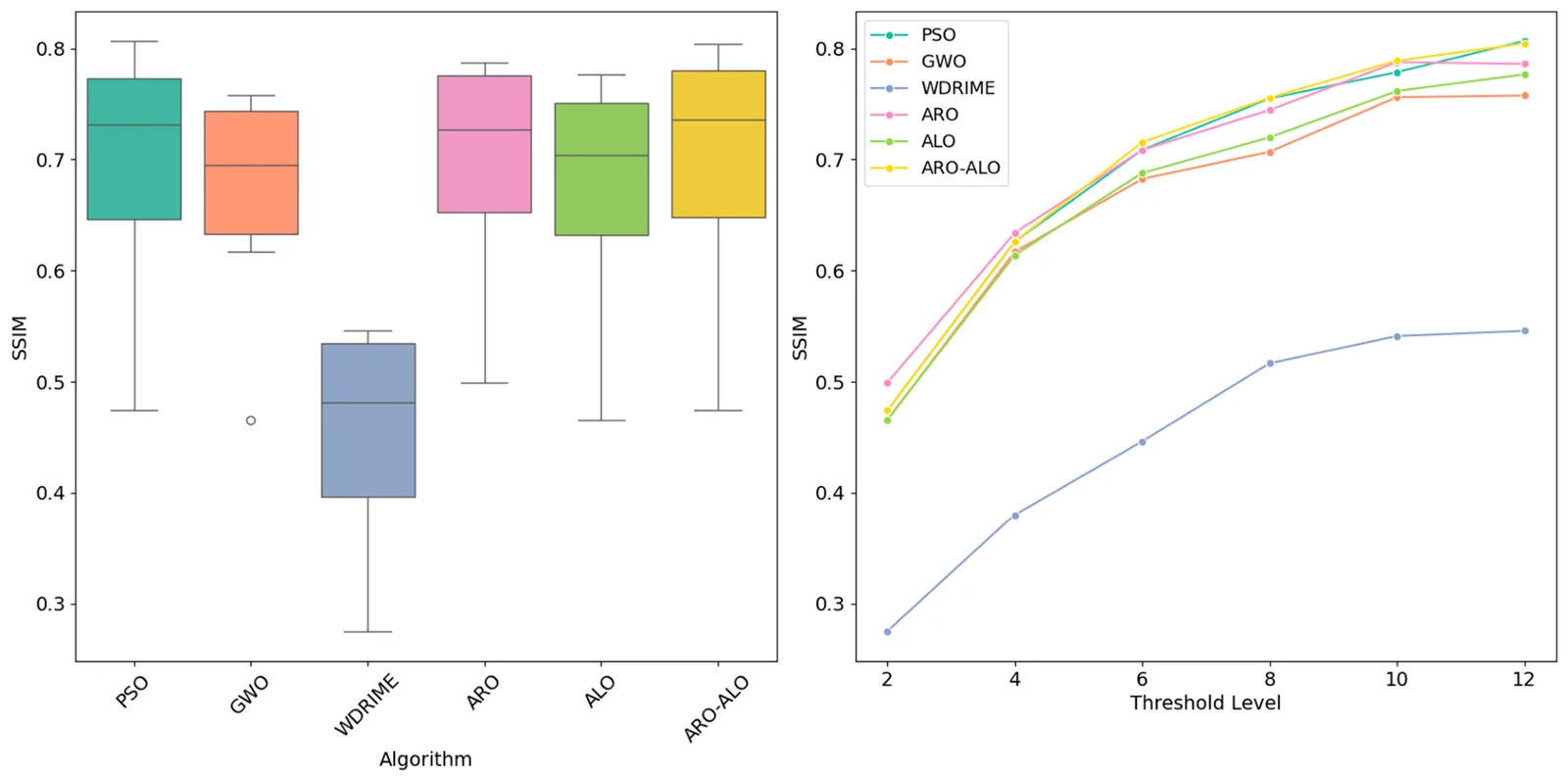
SSIM performance for colon adenocarcinomas images according to algorithms.

When evaluated based on the SSIM metric, the methods are detailed in Table 5 and the comparative graph is presented in Figure 4. At the T = 2 level, the ARO-ALO algorithm demonstrates performance similar to the PSO (0.4741 ± 0.0761) and GWO (0.4656 ± 0.0984) algorithms, achieving an SSIM value of 0.4741 ± 0.0761. However, the ARO (0.4991 ± 0.0897) and ALO (0.4658 ± 0.0987) algorithms show slightly lower values. At the T = 4 level, the ARO-ALO algorithm achieves an SSIM value of 0.6260 ± 0.0426, similar to the PSO (0.6260 ± 0.0426) and GWO (0.6168 ± 0.0547) algorithms. The ARO (0.6339 ± 0.0518) and ALO (0.6139 ± 0.0371) algorithms also produce similar results. At the T = 8 level, the ARO-ALO algorithm achieves a superior SSIM value of 0.7555 ± 0.0801, outperforming the PSO (0.7549 ± 0.0748), GWO (0.7069 ± 0.0745), and WDRIME (0.5166 ± 0.1105) algorithms in structural similarity. Additionally, the ARO (0.7445 ± 0.0815) and ALO (0.7199 ± 0.0765) algorithms demonstrate efficient performance at this level as well. At the T = 10 level, the ARO-ALO algorithm achieves an SSIM value of 0.7889 ± 0.0753, which is more efficient in terms of structural similarity compared to PSO (0.7786 ± 0.0766) and GWO (0.7561 ± 0.0772). Furthermore, the ARO (0.7876 ± 0.0853) and ALO (0.7616 ± 0.0819) algorithms perform slightly lower. At the T = 12 level, the ARO-ALO algorithm demonstrates superior performance with an SSIM value of 0.8043 ± 0.0821, improving segmentation accuracy compared to PSO (0.8067 ± 0.0781) and GWO (0.7576 ± 0.0592). The ARO (0.7860 ± 0.0932) and ALO (0.7766 ± 0.0657) algorithms also exhibit lower performance at this level.

**Figure 5:**
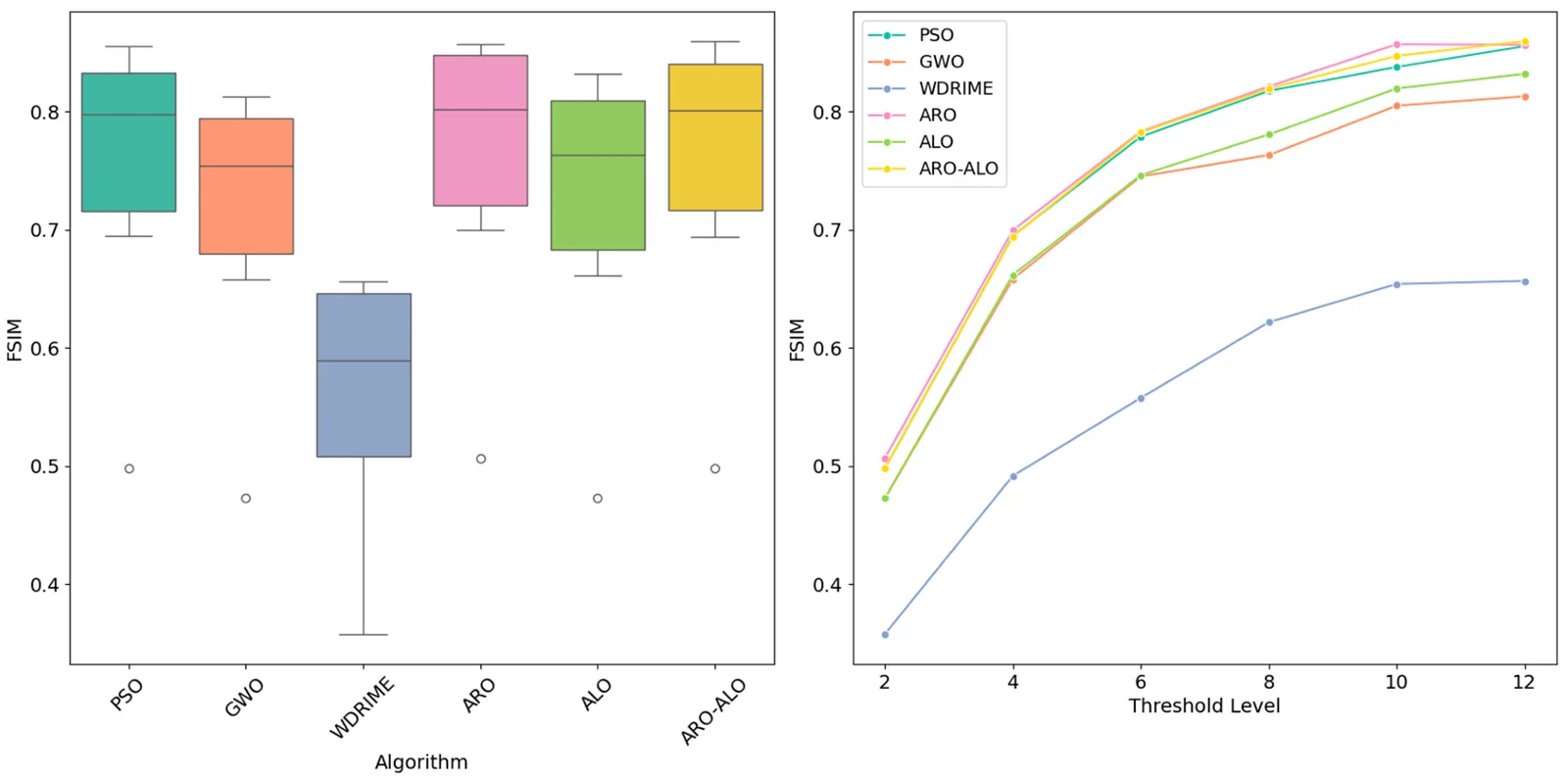
FSIM performance for colon adenocarcinomas images according to algorithms.

The results of the methods in terms of the FSIM metric are presented in detail in Table 6, with the comparative graph shown in Figure 5. At the T = 2 level, the ARO-ALO algorithm demonstrates similar performance to PSO (0.4979 ± 0.0425) and GWO (0.4726 ± 0.0304) algorithms, with an FSIM value of 0.4979 ± 0.0425. The ARO (0.5066 ± 0.0495) and ALO (0.4727 ± 0.0306) algorithms also yield similar results. At the T = 4 level, the ARO-ALO algorithm provides an FSIM value of 0.6941 ± 0.0665, similar to the PSO (0.6947 ± 0.0661) and GWO (0.6582 ± 0.0695) algorithms. The ARO (0.6994 ± 0.0789) and ALO (0.6616 ± 0.0623) algorithms also produce comparable results. At the T = 8 level, the ARO-ALO algorithm achieves a more efficient FSIM value of 0.8192 ± 0.0683 compared to PSO (0.8173 ± 0.0644) and GWO (0.7631 ± 0.0747). WDRIME (0.6216 ± 0.0745), ARO (0.8211 ± 0.0718), and ALO (0.7805 ± 0.0592) algorithms also provide efficient results at this level. At the T = 10 level, the ARO-ALO algorithm enhances segmentation quality with a more efficient FSIM value of 0.8470 ± 0.0607, outperforming PSO (0.8376 ± 0.0559) and GWO (0.8048 ± 0.0561) algorithms in feature similarity. The ARO (0.8568 ± 0.0562) and ALO (0.8194 ± 0.0526) algorithms also show similar performance. At the T = 12 level, the ARO-ALO algorithm yields an FSIM value of 0.8595 ± 0.0578, demonstrating more efficient results than PSO (0.8552 ± 0.0574) and GWO (0.8128 ± 0.0437) algorithms. The ARO (0.8564 ± 0.0650) and ALO (0.8318 ± 0.0407) algorithms are also efficient at this level, but less so. Overall, the ARO-ALO algorithm provides high segmentation accuracy, structural similarity, and content preservation across all threshold levels, and it demonstrates more efficient performance at higher threshold values, improving segmentation accuracy with consistency. This efficient performance indicates that ARO-ALO contributes significantly to improving segmentation accuracy.

#### 4.2.2. Results for Colon Benign Tissue Class

**Figure 6:**
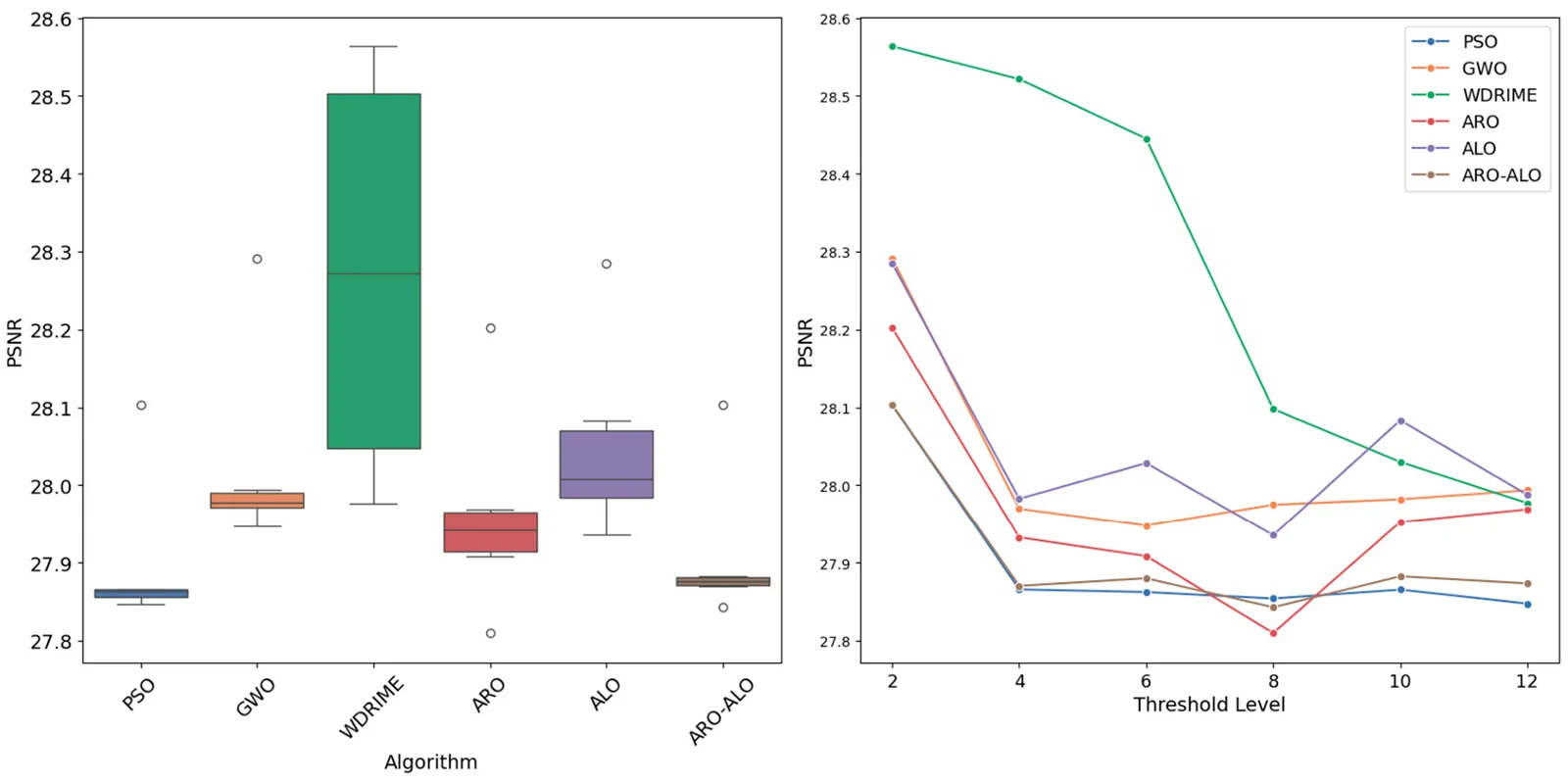
PSNR performance for benign tissue images according to algorithms.

The comparative results of the algorithms in terms of PSNR for colon benign tissue images are summarized in Table 7 and Figure 6. Accordingly, at the T = 2 level, the ARO-ALO algorithm (28.1031 ± 0.4673 dB) yields performance comparable to PSO (28.1031 ± 0.4673 dB) and GWO (28.2912 ± 0.4355 dB), while WDRIME (28.5635 ± 0.3005 dB) achieves the most efficient PSNR value. At the T = 4 level, ARO-ALO (27.8703 ± 0.2606 dB), PSO (27.8657 ± 0.2544 dB), and GWO (27.9696 ± 0.2404 dB) provide similar results, while WDRIME (28.5214 ± 0.3066 dB) again yields a higher PSNR. The lower standard deviation of ARO-ALO makes it a more stable option. At the T = 6 level, ARO-ALO (27.8799 ± 0.2846 dB), PSO (27.8623 ± 0.3224 dB), and GWO (27.9477 ± 0.3004 dB) show similar results, but WDRIME (28.4450 ± 0.4799 dB) provides a higher PSNR. However, the ARO-ALO algorithm produces more reliable results due to its lower variance. At the T = 8, T = 10, and T = 12 levels, ARO-ALO (28.1031 ± 0.4673 dB and 28.8734 ± 0.3723 dB) consistently provides stable PSNR values at each level. However, WDRIME yields slightly more efficient PSNR results at each level, making it a high-performance method. In contrast, the ARO-ALO algorithm ensures more stable results with lower variance.

**Figure 7:**
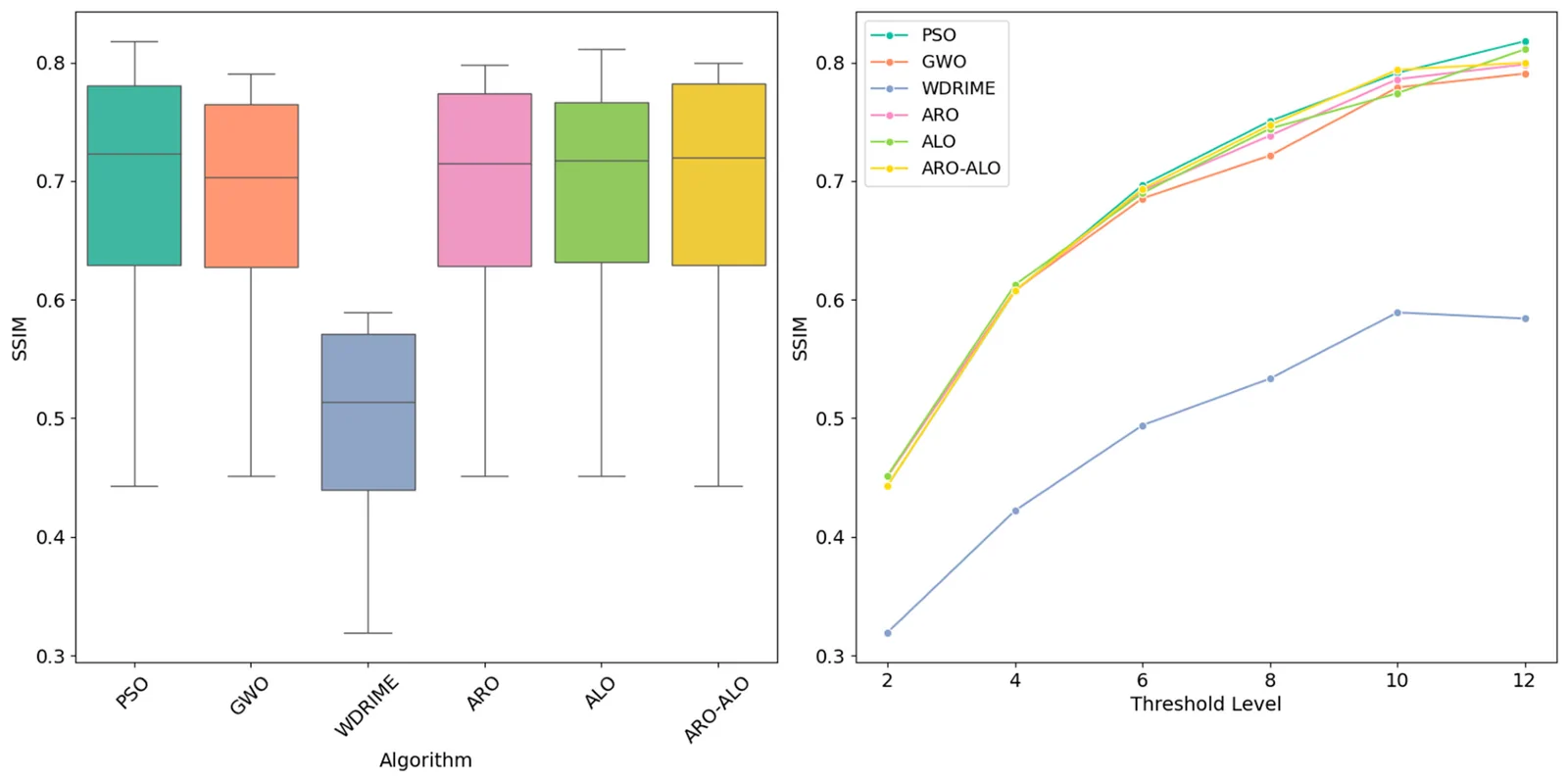
SSIM performance for benign tissue images according to algorithms.

At the T = 2 level, ARO-ALO (0.4431 ± 0.0228) produces similar SSIM values to PSO (0.4431 ± 0.0228) and GWO (0.4509 ± 0.0117), while WDRIME (0.3193 ± 0.0199) shows a significantly lower SSIM value. Here, ARO-ALO provides lower variance and more consistent structural similarity. At the T = 4 level, ARO-ALO (0.6075 ± 0.0307) achieves similar results to PSO (0.6071 ± 0.0320) and GWO (0.6079 ± 0.0398), whereas WDRIME (0.4220 ± 0.0351) exhibits a noticeably lower SSIM value. ARO-ALO offers a more stable and consistent structural similarity. At the T = 6 level, ARO-ALO (0.6933 ± 0.0306) produces similar SSIM values to PSO (0.6964 ± 0.0299) and GWO (0.6853 ± 0.0357), but ARO-ALO provides a more stable result with consistent structural similarity. At the T = 8, T = 10, and T = 12 levels, ARO-ALO (0.7998 ± 0.0330) yields more consistent results with the lowest variance. WDRIME’s lower SSIM values result in less stable performance. The SSIM metric results of the methods are presented in Table 8, with the comparative graph shown in Figure 7.

**Figure 8:**
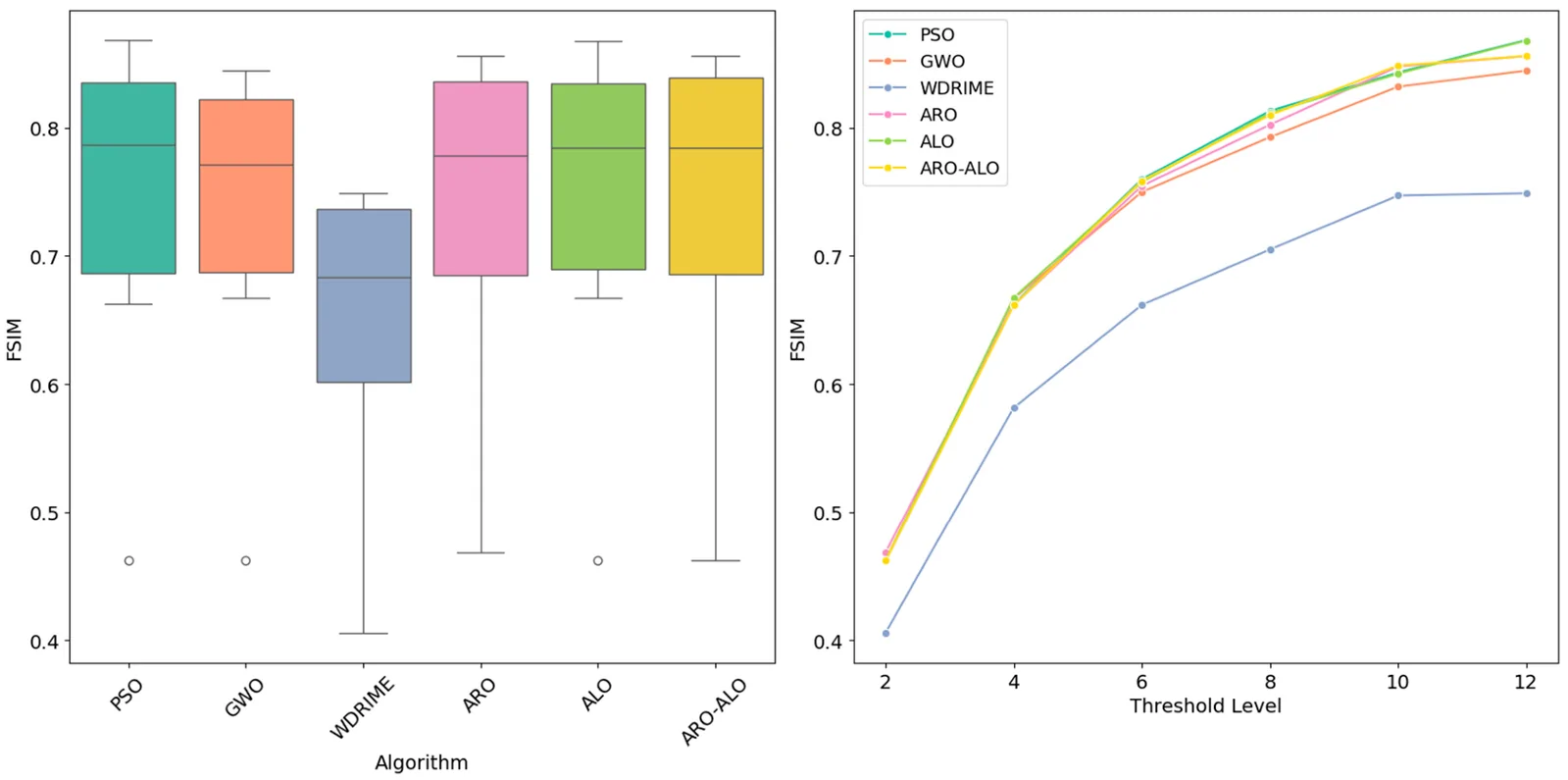
FSIM performance for benign tissue images according to algorithms.

FSIM metrics at the T = 2 level, ARO-ALO (0.4616 ± 0.0292) produces similar results to GWO (0.4618 ± 0.0307), while PSO (0.4616 ± 0.0292) yields the same value. ARO-ALO, in this case, also achieves a more stable result. At the T = 4 level, ARO-ALO (0.6615 ± 0.0287) provides results similar to PSO (0.6623 ± 0.0282) and GWO (0.6668 ± 0.0279), whereas WDRIME (0.5814 ± 0.0314) shows a lower FSIM value. The ARO-ALO algorithm achieves more reliable results with lower variance. At the T = 6 level, ARO-ALO (0.7586 ± 0.0295) produces results similar to PSO (0.7599 ± 0.0300) and GWO (0.7499 ± 0.0331), with ARO-ALO offering a more consistent performance due to its lower variance. At the T = 8, T = 10, and T = 12 levels, ARO-ALO (0.7998 ± 0.0330 and 0.8561 ± 0.0229) presents stable results with high FSIM values. WDRIME, however, performs worse at each level, highlighting the advantage of ARO-ALO. In conclusion, the ARO-ALO algorithm demonstrates comparable or superior performance to PSO and GWO in all metrics. While the WDRIME algorithm performs well only in the PSNR metric, ARO-ALO consistently offers more reliable, stable, and practically effective results across all metrics with lower variance. The FSIM metric results for the methods are presented in Table 9, with the comparative graph shown in Figure 8.

#### 4.2.3. Spatial Correlation and Histogram-Based Threshold Location Analysis

**Figure 9:**
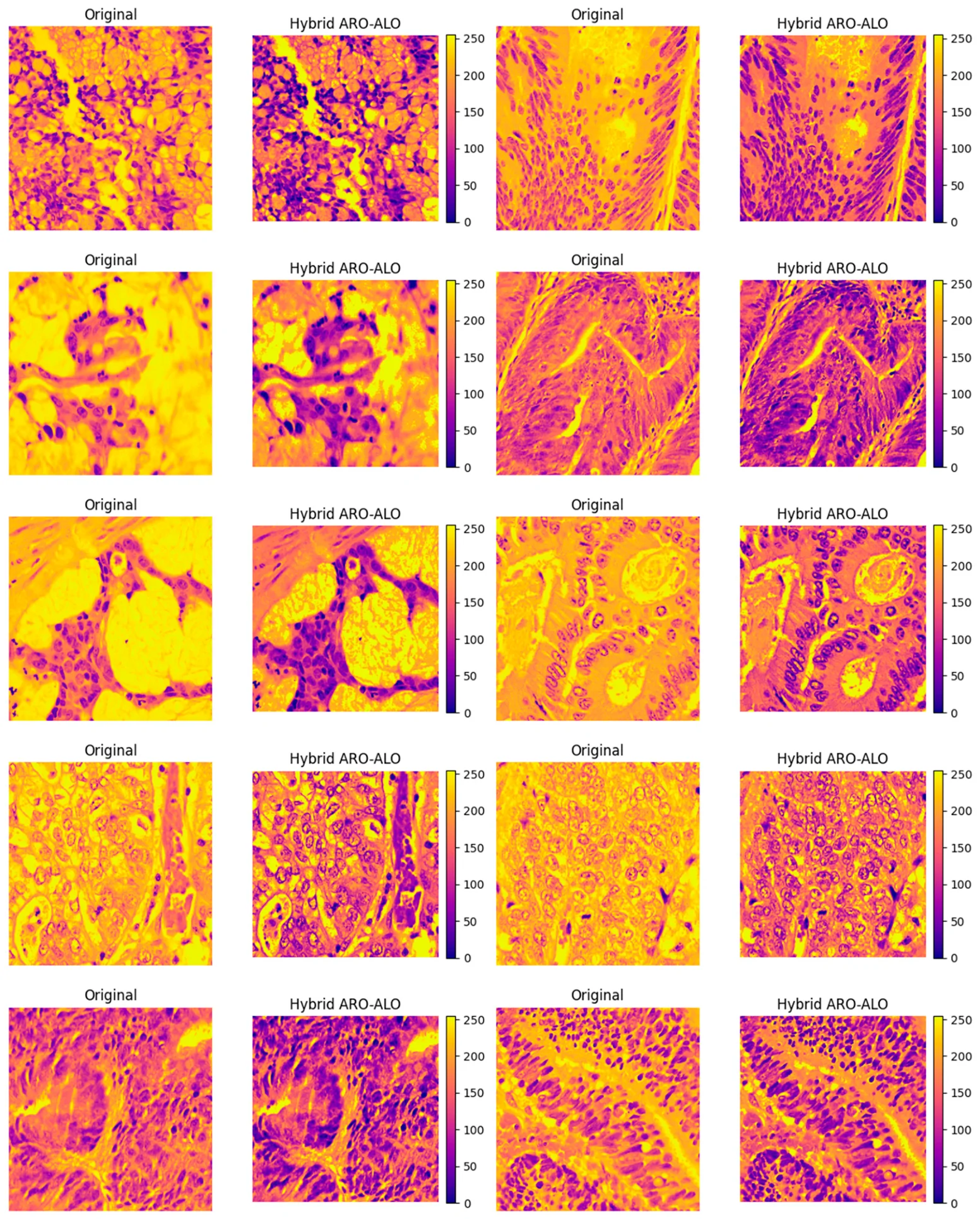
Visual segmentation outputs of test images for the Adenocarcinoma class.

**Figure 10:**
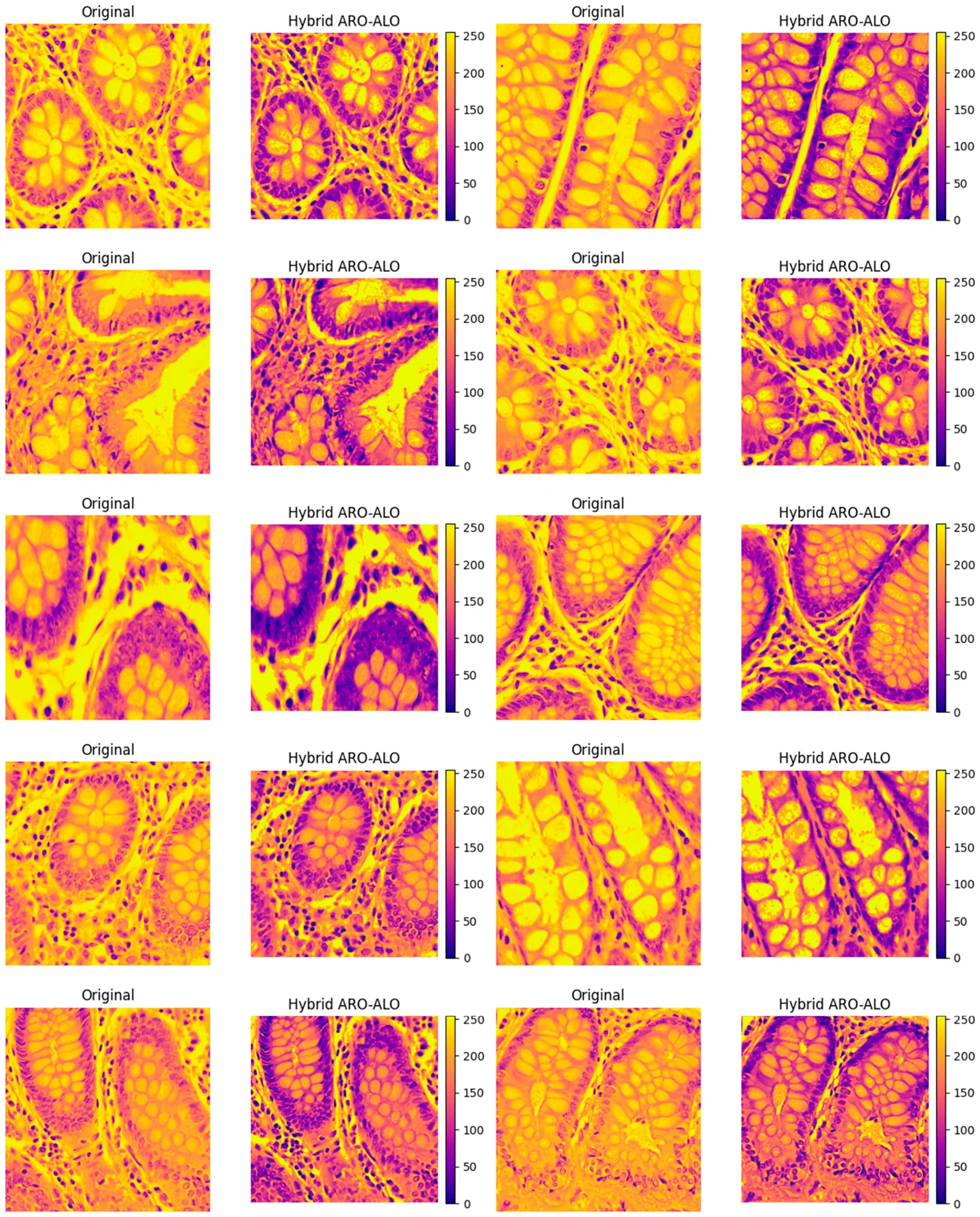
Visual segmentation outputs of test images for the benign tissue class.

The average and standard deviation (Std.) values for the PSNR, SSIM, and FSIM metrics obtained by different algorithms for the Adenocarcinoma and Benign Tissue classes are presented in Table 10. The standard deviation reflects the stability of the algorithms, showing the consistency of the results obtained by each method. For Colon Adenocarcinoma Images, the PSO algorithm demonstrates efficient performance with average values of PSNR: 27.7599, SSIM: 0.6955, and FSIM: 0.7556. However, the high standard deviations in PSNR and SSIM (0.2583 and 0.064) limit the algorithm’s stability. The GWO algorithm exhibits similar performance with PSNR: 27.8882, SSIM: 0.6715, and FSIM: 0.7211. However, higher standard deviations in SSIM and FSIM (0.2711, 0.065, and 0.058) negatively affect the stability of GWO. The WDRIME algorithm achieves the highest PSNR of 28.3458, but with lower visual quality values in SSIM (0.4491) and FSIM (0.5586), it falls behind other algorithms. Additionally, the high standard deviation of WDRIME (0.4495) adversely impacts its stability. The ARO algorithm shows a balanced performance with PSNR: 27.6812, SSIM: 0.6975, and FSIM: 0.7626, exhibiting high stability. The low standard deviations (0.2311, 0.077, and 0.069) increase the reliability of the ARO algorithm. The ALO algorithm demonstrates balanced performance with PSNR: 27.8772, SSIM: 0.6757, and FSIM: 0.7265, but the higher standard deviations in SSIM and FSIM (0.2611, 0.068, and 0.057) limit its stability. The ARO-ALO hybrid algorithm achieves similar results to the ARO algorithm with PSNR: 27.7549, SSIM: 0.6986, and FSIM: 0.7579. However, ARO-ALO improves segmentation performance with higher SSIM values (0.6986) and similar FSIM values. The low standard deviations (0.2615, 0.064, and 0.062) make ARO-ALO more stable and efficient compared to other algorithms. For Colon Benign Tissue Images, the PSO algorithm shows good performance with PSNR: 27.9010, SSIM: 0.6897, and FSIM: 0.7429. However, the high standard deviation in PSNR (0.3241) may negatively impact stability. The GWO algorithm yields similar results with PSNR: 28.0103, SSIM: 0.6779, and FSIM: 0.7329. However, the lower SSIM and FSIM values of GWO limit the visual quality and stability of the algorithm. The WDRIME algorithm achieves a high PSNR value of 28.2861, but with lower visual quality values in SSIM (0.4951) and FSIM (0.6512), it lags behind other algorithms. Furthermore, the higher standard deviations in WDRIME (0.3697 and 0.039) negatively affect its stability. The ARO algorithm demonstrates consistent and stable performance with PSNR: 27.9535, SSIM: 0.6843, and FSIM: 0.7402, exhibiting high stability. The low standard deviations (0.2263, 0.027, and 0.028) enhance stability. The ALO algorithm shows similar performance with PSNR: 28.0380, SSIM: 0.6870, and FSIM: 0.7437, and the low standard deviation in SSIM (0.0257) improves stability. The ARO-ALO hybrid algorithm demonstrates more stable performance compared to ARO and ALO, with PSNR: 27.9049, SSIM: 0.6876, and FSIM: 0.7419, improving stability. The low standard deviations (0.3266, 0.029, and 0.026) enhance the reliability of the ARO-ALO algorithm. The multi-level thresholding performance of the proposed ARO-ALO algorithm on Colon Cancer Histopathology Images is presented in Figure 9 for the colon adenocarcinoma class and in Figure 10 for the benign colon tissue class. Upon examining the images, it is observed that the algorithm creates a clear contrast difference between cellular structures and the background, effectively isolating the cell nuclei. The sharp boundaries in color distribution demonstrate that the algorithm accurately represents different tissue classes. These results show that the proposed ARO-ALO algorithm has a detail-preserving, noise-resistant, and effective segmentation capability on colon cancer histopathology data.

**Figure 11:**
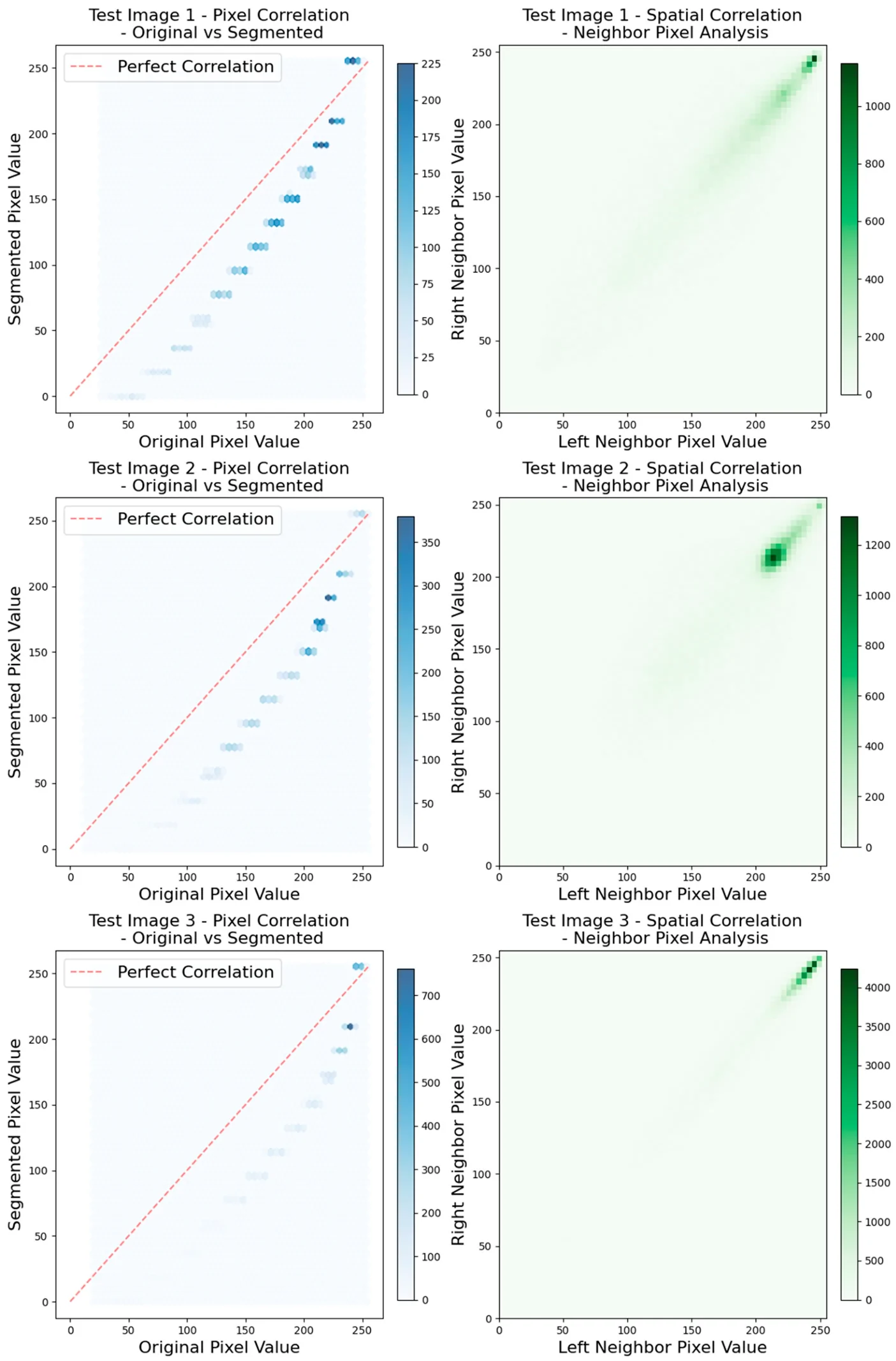

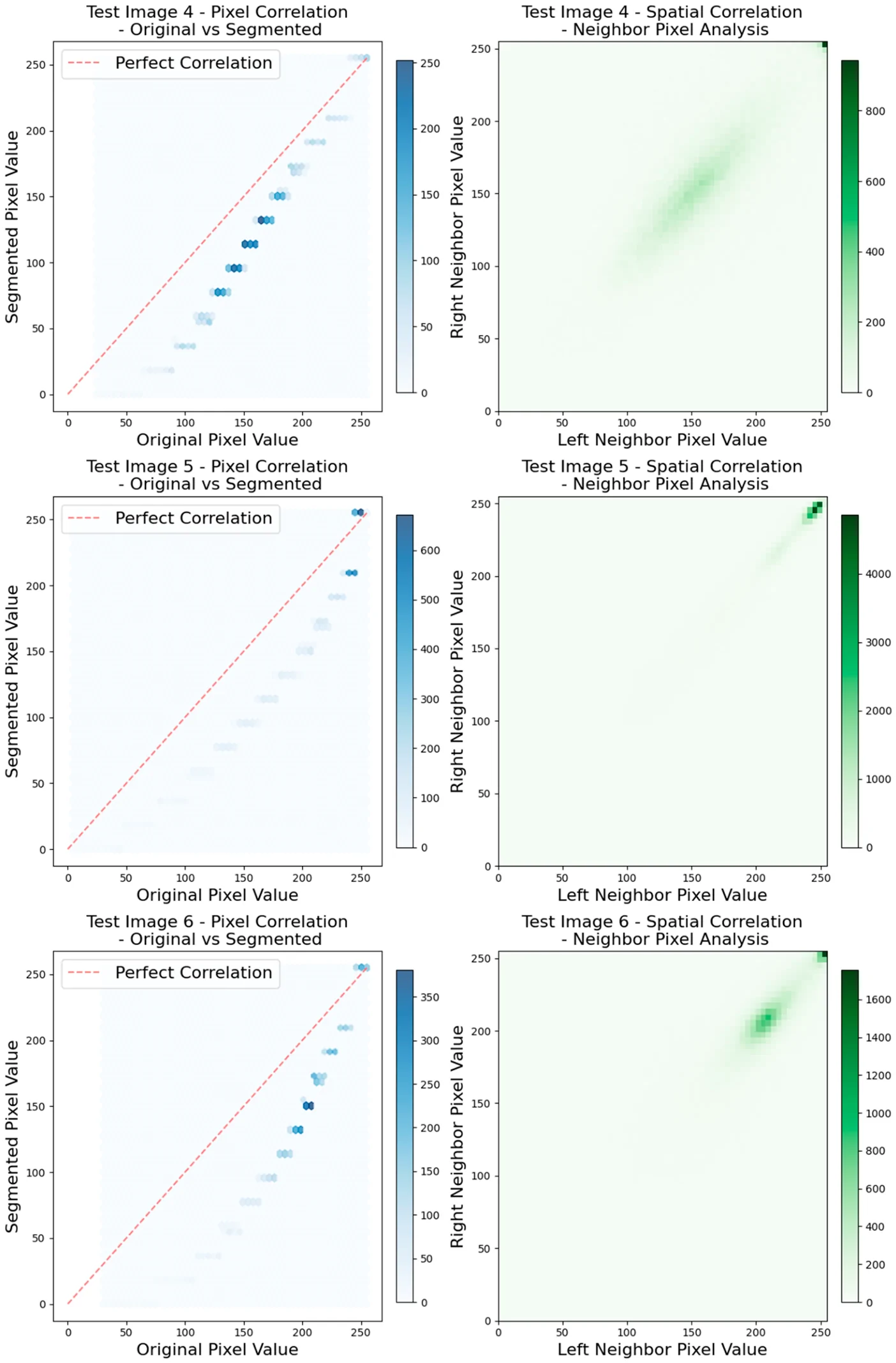
Spatial correlation and neighboring pixel analysis on test images for the Adenocarcinoma class.

**Figure 12:**
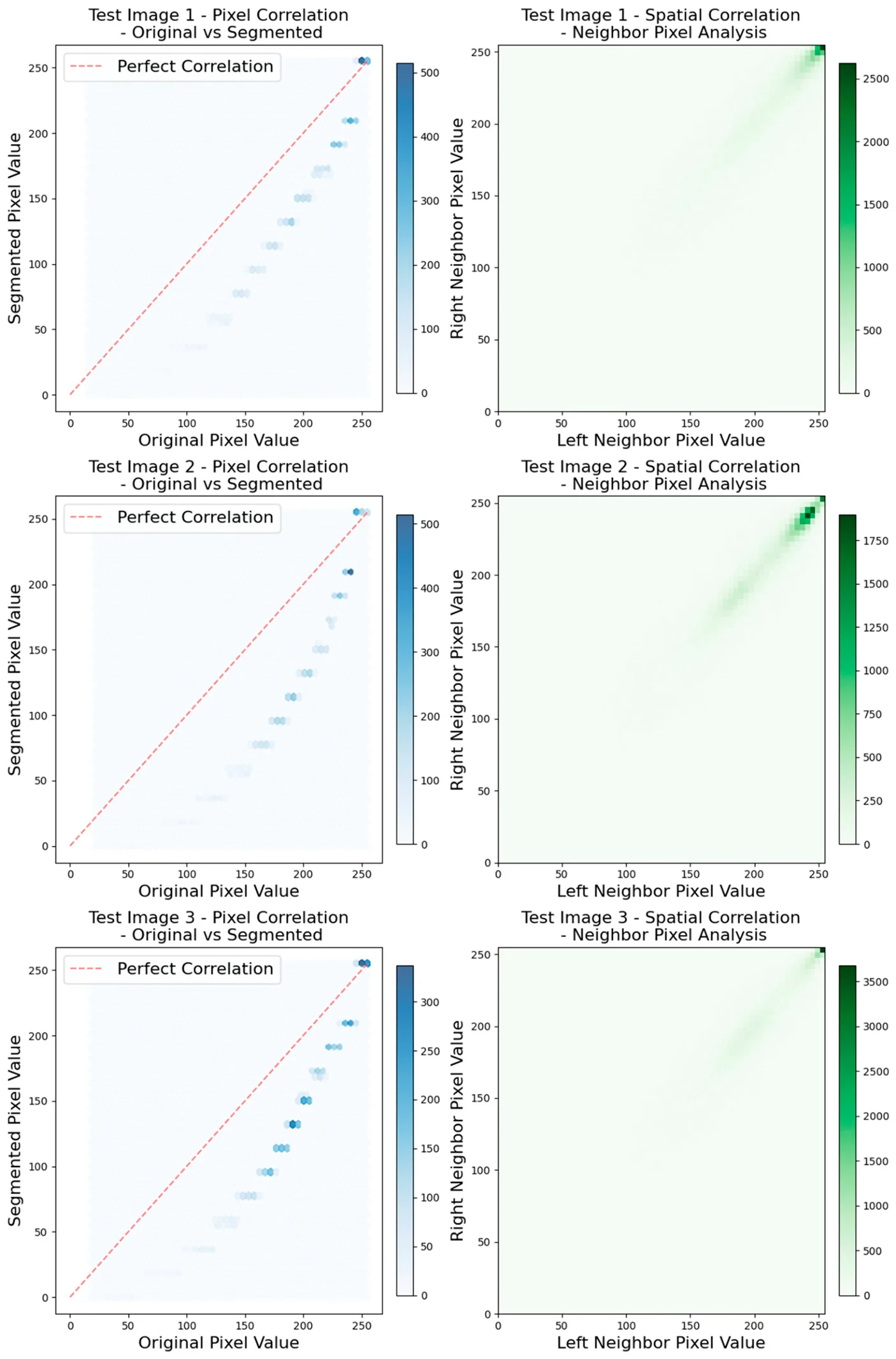

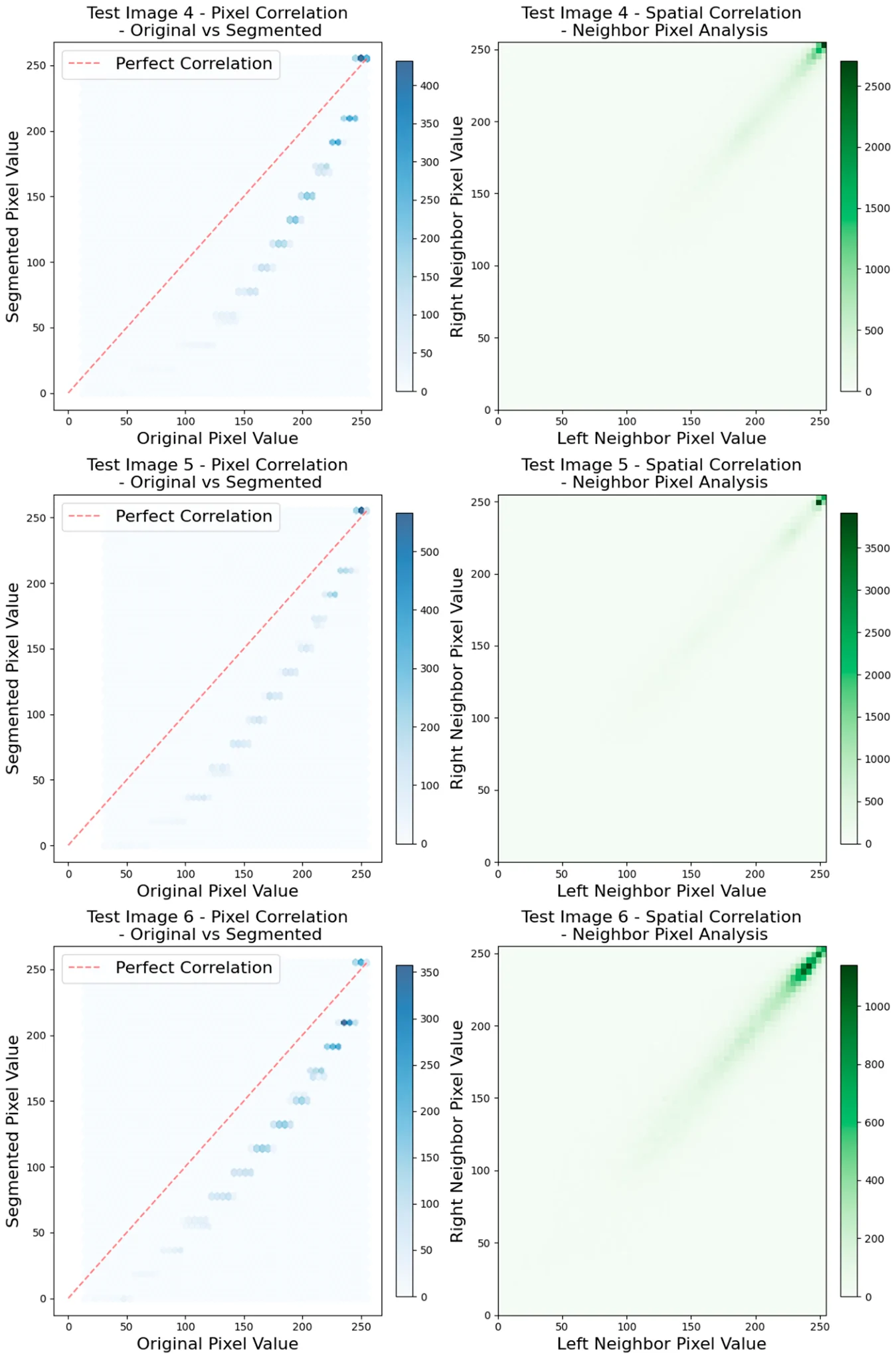
Spatial correlation and neighboring pixel analysis on test images for the benign tissue class.

Additionally, to assess the diagnostic information preservation level of the proposed ARO-ALO algorithm after segmentation, pixel-based and spatial statistical analyses are conducted and presented in Figure 11 and Figure 12. In the Adenocarcinoma class, the Pearson correlation between the original and segmented images is 0.9870, and the Spearman correlation is 0.9948. These results demonstrate that the linear and rank-based relationships between pixel intensities are almost fully preserved, indicating that the proposed algorithm maintains critical image features, such as global contrast, brightness, and color distribution, without distortion. Furthermore, the neighbor pixel analysis reveals the spatial dependency between adjacent pixels, with Pearson and Spearman correlation coefficients of 0.8686 and 0.8653, respectively. These values indicate a strong positive relationship, confirming that the structural integrity of the segmented image is effectively maintained with the proposed method. Thus, the ARO-ALO algorithm not only efficiently thresholds pixel intensities but also preserves tissue continuity and cellular morphology. The obtained correlation coefficients show that the segmentation process is reliable in terms of diagnostic content, which is of great importance for the accurate interpretation of histopathological images. The preservation of boundaries between cell nuclei and cytoplasm, as well as the structural relationships within the tissue, is crucial for pathologists and enhances the clinical applicability of the proposed method. For the Benign Tissue class, the Pearson correlation between the original and segmented images is 0.9692, and the Spearman correlation is 0.9939. These findings confirm that the pixel intensity relationships are well-preserved after segmentation and validate that the proposed algorithm effectively maintains fundamental image features such as contrast, brightness, and color distribution. Additionally, the neighbor pixel analysis for the benign tissue class yields Pearson and Spearman correlation values of 0.8194 and 0.8197, respectively, indicating a strong positive spatial relationship between adjacent pixels. This suggests that the segmentation process preserves the integrity of the tissue structure while providing high diagnostic consistency. Therefore, the ARO-ALO algorithm not only improves pixel intensity thresholding but also preserves tissue continuity and cellular morphology. The analysis demonstrates that the segmentation process is diagnostically reliable, and the main tissue structures are efficiently preserved for accurate pathological evaluation.

**Figure 13:**
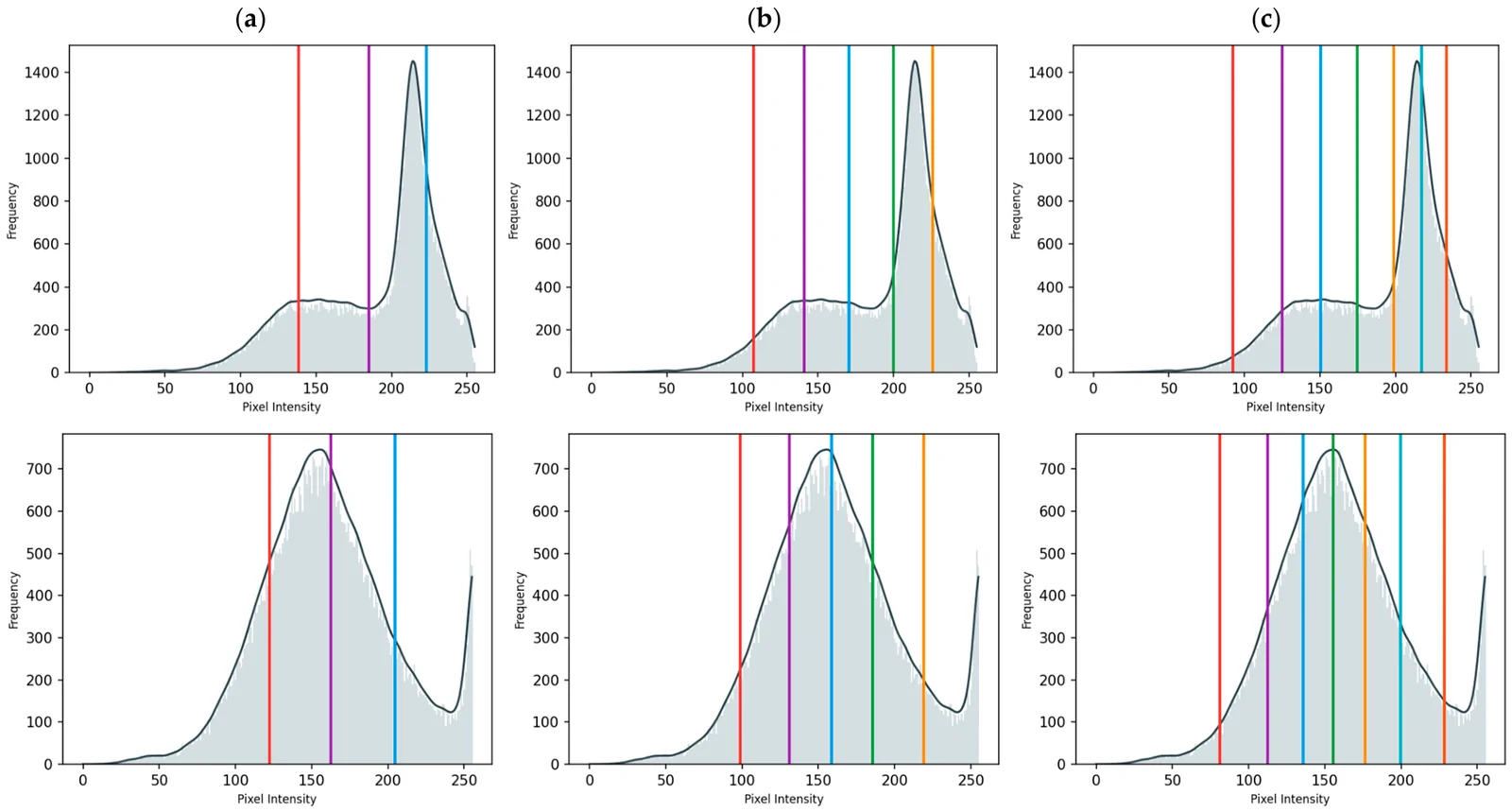

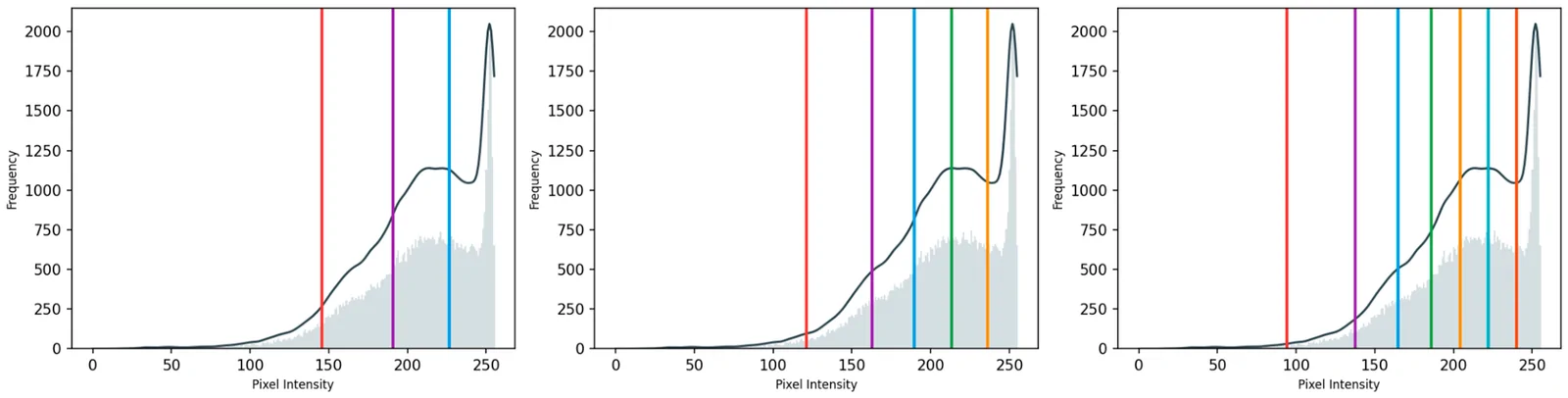
Performance of the proposed ARO-ALO algorithm in threshold setting and intensity valley detection on heterogeneous histopathological tissues. (The subplots in the figure represent threshold levels of (**a**) T = 3, (**b**) T = 5, and (**c**) T = 7 from left to right; the vertical lines indicate the global optimal threshold values set by the algorithm to isolate cellular density clusters).

Beyond quantitative metrics and spatial correlation analyses, visualizing the multi-level thresholding stability of the proposed ARO-ALO algorithm within the context of intensity distributions constitutes a critical requirement for demonstrating the physical rationale of the method on histopathological tissue structures. In multi-level thresholding problems, segmentation errors primarily arise not from the spatial mislabeling of pixels, but rather from the inability of optimization algorithms to correctly identify the natural intensity valleys within the histogram, thereby assigning thresholds to false local minima or peak slopes. Accordingly, in order to intuitively analyze how the proposed method separates cellular morphologies, the grayscale histograms of the test images together with the algorithmically positioned threshold values are presented in Figure 13. Each visual analysis in the figure represents the optimization behavior at threshold levels of T = 3, T = 5, and T = 7, respectively, from left to right. Due to the intrinsic nature of histopathological images, lumen regions, stromal tissues, epithelial cell nuclei, and background noise are highly intertwined, resulting in indistinct, multi-modal, and asymmetric intensity distributions. Within such heterogeneous intensity profiles, increasing the number of thresholds exponentially enlarges the complexity of the search space, thereby increasing the tendency of optimization algorithms to undergo premature convergence by being misled by minor frequency fluctuations around histogram peaks. As observed in the left panels corresponding to the basic thresholding case (T = 3), the identification of dominant valleys is relatively straightforward, whereas the optimization difficulty reaches its maximum level in the middle (T = 5) and right (T = 7) panels as the algorithm attempts to capture the finer structures of the histogram. Nevertheless, despite the increased threshold number (T = 7) and the progressively narrowed class intervals, the ARO-ALO algorithm efficiently positions the threshold boundaries, indicated by vertical lines, onto the deepest and most representative valleys that isolate the principal intensity clusters. The ability of the algorithm to continuously separate the major histopathological classes without being trapped by minor noisy peaks along the histogram slopes physically validates that the switching ratio (λ=0.6), discussed in Section 4.4, effectively establishes a balance between exploration and exploitation. Accurate valley localization maximizes the between-class variance of pixels while simultaneously preventing structural distortions caused by erroneous pixel assignments along cellular tissue boundaries. The observed visual and statistical consistency clearly demonstrates that the superior FSIM and SSIM values achieved by ARO-ALO are not coincidental; rather, they provide strong evidence that the proposed method adapts to the natural intensity hierarchy of histopathological images with high stability and robustness.

### 4.3. Generalization Study on Diverse Histopathological Datasets with Deep Learning and SOTA Metaheuristics

Demonstrating that the proposed ARO-ALO hybrid algorithm does not merely overfit to a specific tissue type (colon adenocarcinoma) but instead possesses a tissue-agnostic generalization capability constitutes a critical requirement for validating the clinical applicability of the proposed methodology. Accordingly, the search behavior and stability performance of the algorithm were evaluated on two independent datasets, namely the Oral Squamous Cell Carcinoma (OSCC) dataset [56] and the Pulmonary Circulation Vessels dataset [57], both of which exhibit cellular morphologies and intensity distributions fundamentally different from colon tissue. The OSCC dataset consists of high-resolution histopathological images obtained from 1325 patients for the diagnosis and prognosis of aggressive oral squamous cell carcinoma. For each patient, the dataset contains six high-resolution images acquired at magnification levels of ×200, ×400, and ×1000, covering both tumor core and edge regions, resulting in a total of 7950 image samples. Since OSCC images contain highly intertwined epithelial–stromal boundaries and heterogeneous cancer cell clusters, they provide an exceptionally challenging analytical framework for evaluating the boundary delineation and clustering sensitivity of optimization algorithms within complex tissue architectures. In contrast, the Pulmonary Circulation Vessels dataset contains 609 pulmonary vessel cross-sectional images and their corresponding masks at a high spatial resolution of 0.68~μm/px. These images specifically target structural deformations such as vascular wall thickening and collagen accumulation within pulmonary arterial branches, thereby enabling the evaluation of the algorithm’s capability to distinguish vascular lumen pixels from vascular wall structures while maintaining robustness against background noise. In this context, whereas the OSCC dataset presents highly intertwined epithelial–stromal structures, the pulmonary dataset introduces challenging microvascular patterns with variable background illumination conditions. Such diversity in tissue morphology establishes a highly demanding analytical benchmark for assessing the noise robustness and histogram valley localization capability of multi-level thresholding algorithms. Furthermore, in addition to the SOTA WDRIME algorithm employed in previous analyses, the proposed framework is also compared against another SOTA hybrid metaheuristic algorithm and a deep learning-based segmentation model. Specifically, the RSA-SSA hybrid architecture [13] and the CUNet3+ [58] deep learning model are benchmarked against the proposed ARO-ALO algorithm. RSA-SSA, formed through the synergistic integration of the RSA and SSA, is regarded as one of the most competitive and contemporary optimization approaches for multi-level thresholding in medical image segmentation. Meanwhile, CUNet3+, equipped with full-scale skip connections and integrated attention mechanisms, is positioned in the literature as a high-performing fully supervised architecture for histopathological segmentation tasks. The experimental analyses presented in this section critically investigate the practical applicability of the proposed unsupervised ARO-ALO algorithm against a label-dependent and computationally intensive deep learning architecture such as CUNet3+. Simultaneously, the numerical findings demonstrate the effectiveness of the transition mechanism (λ=0.6) in enabling the algorithm to locate the global optimum without becoming trapped in local minima, even when compared against a SOTA optimization framework such as RSA-SSA. The generalization capability and stability of the proposed ARO-ALO algorithm across datasets with distinct histopathological characteristics are comprehensively presented through the quantitative analyses summarized in Table 11 and Table 12. The Adenocarcinoma and Benign tissue classes of the LC25000 dataset, used in the primary analyses, further validate the structural preservation efficiency of ARO-ALO when compared with the contemporary SOTA RSA-SSA model. In the Adenocarcinoma class at the threshold level of T = 12, ARO-ALO (SSIM: 0.8043, FSIM: 0.8595) achieves superior performance compared to RSA-SSA (SSIM: 0.8021, FSIM: 0.8557). Similarly, in the Benign tissue class, ARO-ALO (SSIM: 0.7998, FSIM: 0.8561) outperforms RSA-SSA (SSIM: 0.7814, FSIM: 0.8398), particularly in terms of structural similarity and feature preservation. This generalization capability remains consistent across external datasets characterized by entirely different cellular topologies. Analyses conducted on the OSCC dataset, which contains highly complex epithelial tissue structures, demonstrate the efficiency of ARO-ALO in locating the global optimum at high threshold levels (T ≥8) without becoming trapped in local optima. Specifically, at the threshold level of T = 12, the proposed ARO-ALO algorithm achieves SSIM and FSIM scores of 0.8690 and 0.9200, respectively, outperforming the SOTA RSA-SSA model (SSIM: 0.8567, FSIM: 0.9080). This notable improvement in FSIM objectively demonstrates the algorithmic sensitivity of the proposed framework in preserving phase congruency and structural details associated with cancerous cellular boundaries. Although both algorithms maintain relatively low standard deviation values, indicating stochastic optimization stability, the peak performance achieved by ARO-ALO confirms its greater robustness against morphological complexity.

The methodological comparison is further extended through experiments conducted on the Pulmonary Circulation Vessels dataset, where fine microvascular structures are segmented. Since this dataset contains pixel-level annotated ground-truth masks, it enables the training of a fully supervised deep learning model (CUNet3+) and allows objective comparisons with unsupervised metaheuristic approaches. Within the metaheuristic comparison, both ARO-ALO and RSA-SSA demonstrate competitive performance. Although RSA-SSA provides a marginal advantage in the FSIM metric at the threshold level of T = 12 (0.8932), the proposed ARO-ALO algorithm (0.8856) preserves its competitive behavior and demonstrates high resistance against structural domain shifts introduced by the dataset. The results presented in Table 12 provide an objective comparison of the structural preservation and boundary delineation capabilities of deep learning-based (CUNet3+) and metaheuristic-based (ARO-ALO) segmentation paradigms using task-specific segmentation performance metrics. Fully supervised deep learning models are trained to assign pixels directly to discrete semantic classes separated by well-defined categorical boundaries. Consequently, evaluating these models solely using information-preservation metrics such as the SSIM and the FSIM, which primarily reward structural fidelity rather than semantic segmentation accuracy, may place supervised deep learning approaches at an inherent disadvantage. To ensure a fair and clinically meaningful comparison, both methods were comprehensively evaluated using the Dice Coefficient, Jaccard Index, Hausdorff Distance, and Boundary F-Score (BF-Score). The experimental results demonstrate that the CUNet3+ model achieved average Dice and Jaccard scores of 0.8743 and 0.8085, respectively, whereas the proposed unsupervised ARO-ALO algorithm attained substantially higher values of 0.9534 for Dice and 0.9125 for Jaccard, indicating superior semantic segmentation accuracy. More importantly, the considerably higher Hausdorff Distance observed for CUNet3+ (33.7639 ± 67.4273) reveals substantial variability in boundary localization, indicating that the model occasionally fails to accurately delineate cellular structures and produces significant outlier errors in certain histopathological images. In contrast, the proposed ARO-ALO algorithm achieved a remarkably low Hausdorff Distance of 5.7445 together with an excellent BF-Score of 0.9559, demonstrating its ability to preserve cellular tissue topology while accurately delineating structural boundaries without introducing morphological distortions. To further investigate the susceptibility of supervised deep learning models to staining and illumination variations, a robustness analysis was performed by applying controlled perturbation variants to the evaluation dataset. Under these perturbation conditions, the performance of the CUNet3+ model deteriorated considerably, exhibiting average reductions of 0.2957 in Dice, 0.3499 in the Jaccard Index, and 0.3148 in BF-Score, while its Hausdorff Distance increased by an average of 60.9402 pixels. These findings indicate that the supervised model largely adapts to the limited staining and illumination characteristics represented in the training dataset and therefore exhibits limited generalization capability when evaluated on previously unseen images acquired under different scanner settings or staining protocols. By contrast, the proposed ARO-ALO algorithm performs dynamic threshold optimization directly on the instantaneous histogram distribution of each input image without relying on annotated training masks. This training-free optimization mechanism enables the algorithm to maintain substantially greater structural stability under perturbation conditions. Specifically, the consistency analysis demonstrates that ARO-ALO preserves robust segmentation performance under image perturbations, achieving Dice and BF-Score consistency values of 0.7166 and 0.7743, respectively, while maintaining a comparatively low Hausdorff consistency value of 31.0658, thereby indicating superior preservation of tissue morphology despite variations in imaging conditions. Overall, these findings suggest that, because ground-truth annotation masks inherently suppress the continuous cellular intensity distributions of histopathological tissues by converting them into homogeneous categorical labels, supervised deep learning models remain susceptible to both intrinsic tissue-information loss (reflected by the low SSIM value of 0.1362) and overfitting-induced generalization limitations. In contrast, the proposed hybrid ARO-ALO architecture effectively adapts to the natural intensity hierarchy of histopathological tissues through optimization rather than label-dependent learning, thereby providing a robust, morphologically faithful, and clinically relevant alternative for histopathological image analysis and CDSSs.

### 4.4. Fair Evaluation Budget and Enlarged Ablation Analysis

**Figure 14:**
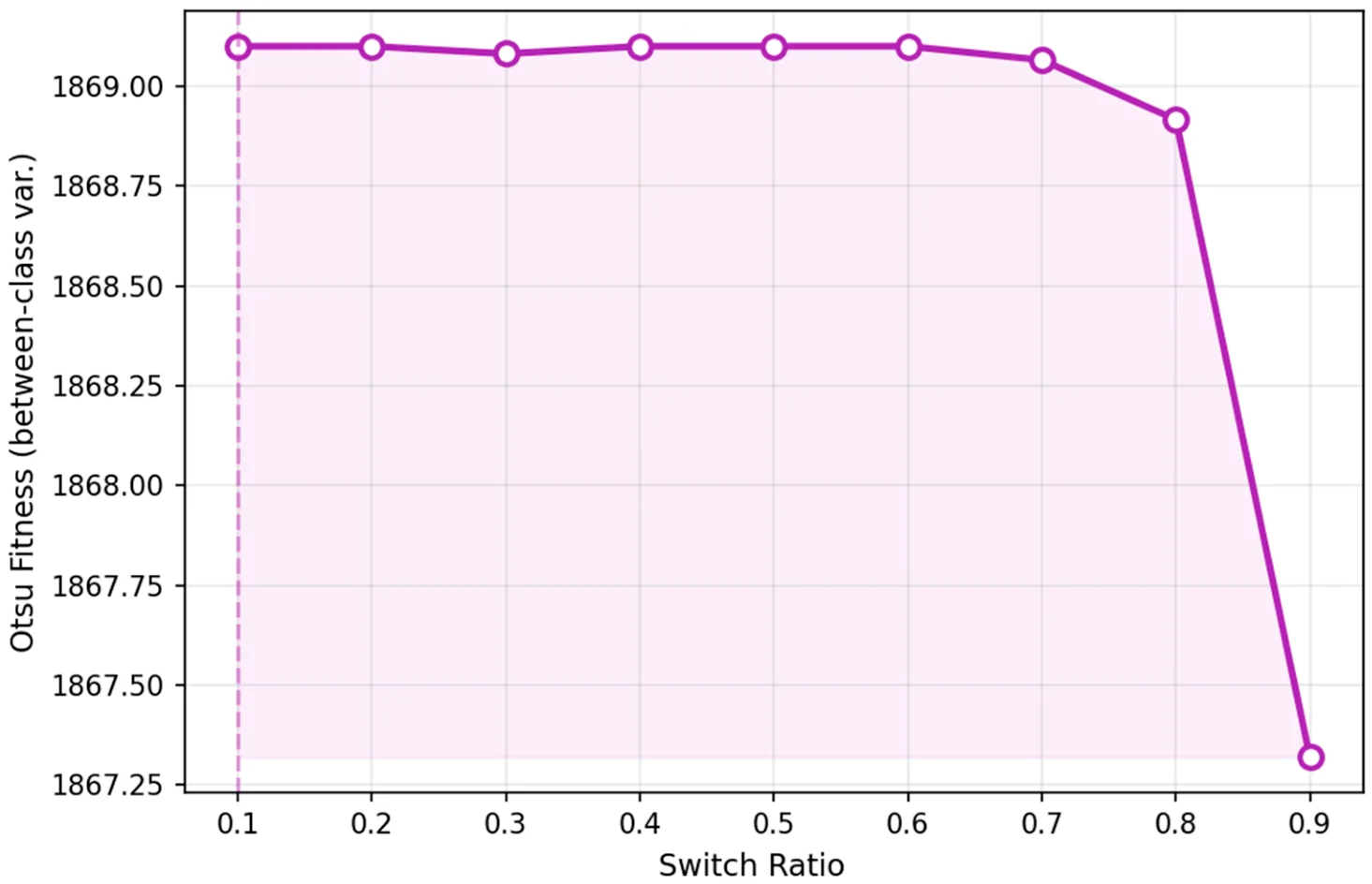
Ablation analysis demonstrating the effect of the λ on the Otsu fitness value in the proposed ARO-ALO hybrid algorithm.

In order to objectively evaluate the design rationale of the proposed ARO-ALO hybrid algorithm and to investigate the structural contributions of its algorithmic components, a comprehensive ablation analysis was conducted using critical control parameters and independent sub-architectures (ARO and ALO). In this context, the effect of the λ, which governs the transition between the exploration and exploitation phases forming the foundation of the algorithm, was analyzed through the Otsu fitness variance graph presented in Figure 14. Accordingly, the λ parameter, representing the fraction of total search iterations allocated to the ARO phase, exerts a direct and asymmetric influence on segmentation quality. When the λ value remains within the range of 0.1–0.7, the algorithm successfully converges to the global optimum while maintaining a highly stable fitness value around the 1869.00 threshold. Nevertheless, enhancing the robustness of the algorithm against general histopathological variations constitutes a critical optimization constraint. Although lower exploration ratios such as λ≤0.4 occasionally produce high Otsu variance values for certain images, the insufficient exploration of the search space increases the risk of becoming trapped in local optima across different tissue morphologies. Conversely, beginning from the threshold of λ≥0.8, and particularly at λ=0.9, a sudden and dramatic decline in the fitness value toward approximately 1867.30 is observed. This sharp structural degradation mathematically demonstrates that allocating 90% of the search iterations to the exploration (ARO) phase restricts the local search and refinement capability of the ALO exploitation mechanism. Insufficient iteration allocation to the exploitation phase prevents the algorithm from adequately intensifying around the identified candidate solution regions (histogram valleys and peaks), thereby leading to premature convergence. Based on these ablation findings, the value of λ=0.6 was not selected arbitrarily; rather, it was fixed as a logically optimized design parameter to ensure an optimal balance between exploration and exploitation. In addition to the rational optimization of the transition parameter, the modular contribution of the hybrid architecture was further validated through comparative statistical analyses performed against the standalone ARO and ALO algorithms. Across both Adenocarcinoma and Benign tissue classes, the standalone ARO and standalone ALO algorithms generated increasing variance at high threshold levels, whereas the proposed ARO-ALO hybrid architecture systematically minimized this variance across PSNR, SSIM, and FSIM metrics. Particularly at high threshold levels (T = 8, 10, 12), the tendency of the individual algorithms to become trapped within optimization bottlenecks was completely mitigated by the stable performance of ARO-ALO characterized by consistently low standard deviation values. Based on the FSIM metrics, the content preservation capability of the ARO-ALO algorithm was found to be substantially more efficient and consistent than those of its independent constituent components. Consequently, the conducted ablation study clearly demonstrates that the high morphological accuracy and stability achieved by ARO-ALO are not coincidental; rather, they arise from a logical synergy established through the fixed-ratio λ parameter, which effectively combines the broad search-space exploration capability of ARO with the precise convergence strength of ALO.

The computational cost and practical applicability of the evaluated algorithms were assessed based on their average execution time per iteration and presented in Table 13. To ensure a hardware-independent and unbiased comparison, all algorithms were evaluated under identical experimental conditions. Among the baseline optimization methods, PSO and GWO exhibited the lowest computational overhead, with average iteration times of 0.0170 s and 0.0168 s, respectively. In contrast, algorithms employing hierarchical or computationally intensive search strategies, namely the hybrid RSA-SSA algorithm and ALO, incurred substantially higher computational costs, requiring average iteration times of 0.2295 s and 0.0564 s, respectively. The proposed ARO-ALO hybrid algorithm achieved an average iteration time of 0.0322 s, demonstrating considerably greater computational efficiency than both the standalone ALO algorithm and the SOTA RSA-SSA approach. These findings indicate that the proposed hybrid framework successfully achieves an optimal balance between computational efficiency and optimization capability, thereby providing a computationally efficient yet highly effective solution for complex optimization tasks.

Comparing optimization algorithms solely on the basis of the number of iterations may obscure differences arising from their population dynamics and internal search mechanisms. To establish a fair and unbiased comparative evaluation, all optimization methods were assessed under an equal Function Evaluation (FE) budget, and the corresponding results are presented in Table 14. Furthermore, to characterize the difficulty of the optimization landscape, the absolute global optimum was determined through Exhaustive Search for low-threshold scenarios (T ≤ 4). In addition, Random Search, the conventional Otsu Dynamic Programming (DP) algorithm, K-Means clustering, and the SOTA general-purpose optimizer CMA-ES were incorporated into the comparative analysis. For the maximization of Otsu’s objective function, namely the between-class variance, Exhaustive Search achieved the absolute global optimum with an objective value of 2502.79. Owing to the combinatorial growth of the search space, Random Search exhibited limited optimization capability, yielding a substantially lower objective value accompanied by a higher standard deviation (±86.26) and degraded structural preservation performance (SSIM = 0.5791). In contrast, the proposed ARO-ALO algorithm achieved an objective value of 2502.58 ± 0.0256, demonstrating near-global optimal convergence with remarkable run-to-run stability. The obtained results further demonstrate that maximizing the mathematical objective function alone does not necessarily guarantee the highest structural fidelity in medical image segmentation, as objectively reflected by the performance distributions of the evaluated algorithms. For example, although the ARO (Partial Budget) algorithm produced the highest SSIM value (0.7048), indicating superior structural preservation, it remained suboptimal in terms of objective function optimization (2499.14) while exhibiting relatively unstable convergence behavior (±2.9070). Conversely, both Exhaustive Search, which attained the absolute global optimum, and the highly stable CMA-ES algorithm (2502.70) achieved excellent optimization accuracy but resulted in partial degradation of tissue topology, yielding an SSIM value of only 0.6849. By comparison, the proposed ARO-ALO algorithm converged substantially closer to the global optimum (2502.58) while simultaneously achieving a higher level of structural preservation (SSIM = 0.6864) than the mathematically optimal solution obtained by Exhaustive Search. These findings indicate that the proposed hybrid architecture establishes an effective balance between objective optimization accuracy and the preservation of cellular morphological fidelity, thereby demonstrating that the mathematically optimal threshold configuration is not necessarily synonymous with the morphologically optimal segmentation. To isolate the unique contribution of the proposed hybridization strategy from the potential confounding effect of an increased optimization budget, an Enlarged Budget ARO ablation study was conducted. Even when the standalone ARO algorithm was allocated the same total FE budget as the proposed hybrid framework, it achieved an objective value of only 2501.33 with a significantly higher run-to-run variability (±1.4627), remaining considerably inferior to the performance of the proposed ARO-ALO architecture (2502.58 ± 0.0256). These findings demonstrate that the superior performance of ARO-ALO cannot be attributed merely to a larger optimization budget; rather, it originates from the mathematically transition mechanism (λ) governing the balance between the exploration and exploitation phases throughout the optimization process.

**Figure 15:**
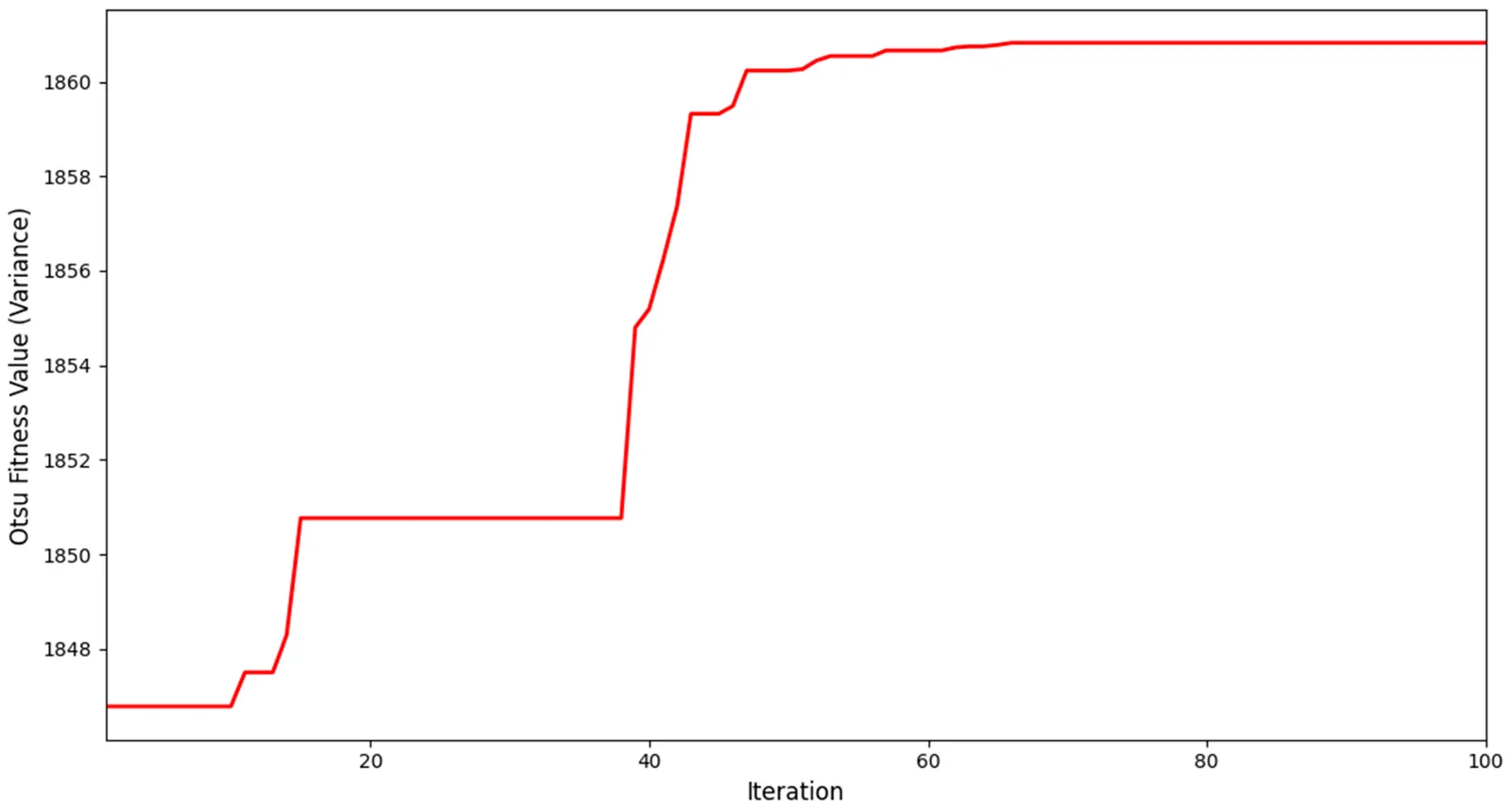
Convergence curve analysis.

In the proposed ARO-ALO algorithm, the selection of the transition parameter (λ), which governs the balance between the exploration and exploitation phases, was determined through a sensitivity analysis conducted on an independent held-out tuning subset that was completely isolated from the test dataset. To objectively evaluate the influence of this parameter on the optimization dynamics, λ was varied over the range of 0.4–0.8, and the corresponding performance results are presented in Table 15. The experimental results indicate that increasing the value of λ, thereby allocating a larger proportion of the optimization budget to the ARO-based exploration phase, produces a nearly monotonic improvement in pixel-level noise suppression performance, as reflected by the PSNR. Specifically, the PSNR increased from 27.2130 dB at λ = 0.4 to 27.3874 dB at λ = 0.8. However, when structural preservation metrics, namely the SSIM and the FSIM, are considered, an inherent trade-off becomes evident. Excessive emphasis on the exploration phase (λ = 0.8) limits the local exploitation capability of the ALO component, resulting in a slight reduction in SSIM to 0.8687 and consequently introducing subtle structural distortions in the cellular topology. Conversely, the configuration with λ = 0.6 achieved the highest FSIM value (0.9135), indicating the most effective preservation of textural characteristics and phase congruency, both of which are critical for reliable histopathological interpretation. Based on these empirical findings, the selected value of λ = 0.6 cannot be regarded as a dataset-specific empirical choice; rather, it represents a globally optimized and fixed hyperparameter that maximizes the balance between pixel-level reconstruction accuracy (PSNR) and morphological preservation (SSIM/FSIM). The convergence curve corresponding to the proposed algorithm is presented in Figure 15. Consistent with the theoretical design of the transition mechanism (λ = 0.6), the stochastic exploration behavior of the ARO component terminates precisely at the 60th iteration, after which the optimization process transitions to the elitist exploitation mechanism of ALO. Following this transition, the convergence curve rapidly stabilizes and approaches a nearly horizontal asymptote, indicating that the algorithm successfully identifies the vicinity of the global optimum without suffering from premature convergence and subsequently performs fine-grained refinement during the local exploitation stage. This convergence behavior provides empirical evidence that the proposed allocation of the iteration budget between the exploration and exploitation phases is appropriately designed to overcome the challenges of the optimization landscape while maintaining stable convergence characteristics.

### 4.5. Statistical Significance Analysis and Method Validation

To verify that the segmentation performance achieved by the proposed ARO-ALO algorithm over both classical and state-of-the-art optimization methods (WDRIME, ARO, ALO, and RSA-SSA) is not attributable to random variation, non-parametric statistical analyses were performed. The corresponding results are summarized in Table 16. Specifically, differences in algorithmic performance and central tendency were evaluated using the Wilcoxon Signed-Rank Test and the Friedman Test with post-hoc multiple comparison correction, considering the comprehensive performance distributions obtained across all threshold levels and benchmark datasets. For the pairwise comparisons, the Wilcoxon Signed-Rank Test was conducted using a significance level of α = 0.05. The statistical analysis revealed that the proposed ARO-ALO algorithm significantly outperformed WDRIME (*p* = 0.001), GWO (*p* = 0.006), ALO (*p* = 0.035), and ARO (*p* = 0.041), with all comparisons yielding statistically significant differences (*p* < 0.05). These results demonstrate the superior optimization effectiveness and run-to-run stability of the proposed hybrid algorithm relative to these competing methods. To assess the overall ranking of the evaluated algorithms, the Friedman test was performed on the repeated-measure performance results. According to the obtained rankings, ARO-ALO achieved the lowest mean rank (1.85), thereby emerging as the best-performing reference method among the evaluated optimization algorithms. Furthermore, based on the Critical Difference (CD) determined by the Nemenyi post-hoc test, the superiority of ARO-ALO over WDRIME, GWO, ALO, and ARO was confirmed to be statistically significant. In contrast, the statistical analyses indicated that RSA–SSA (*p* = 0.082) and PSO (*p* = 0.384) belong to the same statistical performance group as ARO-ALO, suggesting that no statistically significant differences were observed between these methods at the selected significance level. Accordingly, the principal methodological contribution of this study is not to claim universal dominance of the proposed algorithm over all competing approaches. Rather, it lies in demonstrating that ARO-ALO consistently achieves segmentation performance comparable to one of the strongest optimization methods reported in the literature, namely RSA-SSA, while simultaneously exhibiting lower variability and greater statistical stability across repeated experimental trials.

To evaluate the morphological preservation capability of the optimization algorithms and assess their statistical consistency, Intraclass Correlation Coefficient (ICC) analyses were performed. The calculated ICC(3,1) value, obtained across different image instances and thresholding configurations, was determined as 0.8270, indicating a strong level of agreement among the evaluated algorithms in terms of their optimization behavior. Furthermore, the evaluation framework was not restricted solely to pixel-level noise-based metrics, such as PSNR; instead, structural similarity measures, including SSIM and FSIM, were incorporated to ensure a multidimensional assessment of segmentation performance. This methodological design enables the effective characterization of cellular integrity (FSIM), structural and textural topology preservation (SSIM), and signal-level deviations (PSNR) as complementary and non-redundant evaluation components. Consequently, the proposed evaluation strategy provides a more comprehensive and reliable assessment of algorithmic performance by simultaneously considering intensity fidelity, structural preservation, and morphological consistency.

## 5. Discussion

In this study, the optimization performance of the proposed ARO-ALO hybrid architecture, developed for the multilevel thresholding problem of histopathological images within high-dimensional search spaces, is comprehensively analyzed through comparative evaluations against conventional algorithms, SOTA optimizers, and modern deep learning paradigms. Although existing algorithms reported in the literature tend to converge toward near-global optima at low threshold levels (T≤4), they may encounter significant difficulties at higher threshold levels (T≥8), where the combinatorial complexity of the search space increases substantially due to the requirement for separating clinically meaningful microstructures, resulting in susceptibility to stagnation in shallow local minima. The limited effectiveness of existing hybrid metaheuristic approaches for such complex histopathological tissue analyses is primarily associated with their inability to sufficiently adapt exploration and exploitation dynamics to asymmetric cellular intensity distributions. Most hybrid frameworks employ static linear control mechanisms to preserve population diversity, which may lead to inefficient utilization of the optimization budget and increased risks of inaccurate pixel assignments, particularly in highly interwoven epithelial–stromal boundaries. The proposed ARO-ALO architecture addresses the premature convergence problem through a phase-transition mechanism governed by the λ parameter. The convergence dynamics empirically demonstrate that ARO initially explores broad regions of the search landscape during early iterations, after which the exploitation phase of ALO becomes dominant and efficiently isolates deeper histogram valleys. This transition mechanism provides the methodological foundation for the consistent performance of the proposed algorithm under high-threshold segmentation scenarios. The preference for unsupervised thresholding approaches over modern supervised or self-supervised deep learning segmentation models is supported by analytical considerations. Supervised networks transform the naturally continuous intensity gradients of medical images into discrete categorical labels imposed by external ground-truth masks, which introduces an inherent limitation in preserving cellular morphology. Comparative analyses conducted between the proposed approach and deep learning-based models using task-specific segmentation metrics, including Dice coefficient, Jaccard index, Hausdorff distance, and BF-Score, indicate that fully supervised networks may exhibit overfitting tendencies toward the specific illumination and staining characteristics represented in their training datasets. While performance degradation was observed for deep learning models under controlled perturbation scenarios, the unsupervised ARO-ALO mechanism remained capable of directly adapting to the instantaneous histogram distribution of input images without dependence on annotated data, thereby providing substantial potential for preserving structural topology in CDSSs. Another common limitation of existing segmentation models is their dependency on specific tissue types. Therefore, the generalization capability of the proposed approach is investigated using both the OSCC and Pulmonary Circulation Vessel datasets. To ensure a fair comparison among optimization algorithms, all experimental evaluations were standardized according to an equal FE budget rather than the number of iterations, which may conceal differences arising from internal search dynamics and population update mechanisms. The Enlarged Budget ARO ablation study performed under an extended FE budget demonstrates that increasing the evaluation budget alone is insufficient to guarantee superior optimization performance. Instead, the morphological preservation capability and computational efficiency of ARO-ALO, including its effective iteration cost, originate from the synergistic interaction between the integrated optimization mechanisms. The limitations and design choices associated with the experimental framework used for algorithmic validation were also critically examined within an analytical context. Prior to segmentation analysis, high-resolution histopathological images were resized to 256×256 pixels. Although this resizing operation may potentially reduce spatial resolution in fine-scale structures such as capillaries, it substantially decreases the computational complexity of histogram-based thresholding and facilitates the practical implementation of evolutionary optimization strategies. Despite this dimensional reduction, the obtained phase congruency (FSIM) and correlation scores confirm that the diagnostic integrity of the images is largely preserved. Furthermore, each of the 100 clinical images used during the testing phase represents an independent, heterogeneous, and high-dimensional optimization problem that must be solved from scratch by the metaheuristic algorithms. Under the equal FE budget constraint, the stochastic stability of the proposed method was not assessed solely through repeated execution counts; instead, it was additionally supported by low variance values and high inter-metric agreement measured through the ICC analysis. This optimization stability was further validated using non-parametric statistical approaches, including the Wilcoxon Signed-Rank Test and the Friedman Test with post-hoc correction, which demonstrated statistically significant performance differences among competing algorithms (p<0.01). In conclusion, the findings obtained from Pearson and Spearman correlation analyses indicate that the proposed ARO-ALO algorithm is capable of preserving diagnostically critical morphological information following cellular segmentation. The statistical relationships observed between the original and segmented histopathological images demonstrate that the proposed framework extends beyond a mathematical optimization improvement; rather, it provides an efficient and morphologically consistent preprocessing strategy capable of supporting supervised segmentation networks in pathological diagnosis and oncological staging workflows.

## 6. Limitation

Although the findings obtained within the scope of this study clearly demonstrate the potential of the proposed ARO-ALO algorithm for histopathological image segmentation, several limitations must be addressed before the methodology can be fully integrated into clinical practice. First, despite the demonstrated generalizability of the algorithm across diverse datasets such as OSCC and Pulmonary Circulation Vessels, the conducted analyses remain limited to patch-based two-dimensional microscopic tissue sections at the cellular level. This limitation introduces significant computational complexity when attempting to directly apply the algorithm to WSIs, which may contain billions of pixels, or to multidimensional tissue scanning frameworks. While deep learning models are capable of performing inference within seconds after the completion of training, metaheuristic algorithms inherently require the optimization process to be restarted iteratively for each new image. Consequently, this iterative optimization requirement constitutes a substantial bottleneck for real-time diagnostic applications. A second limitation concerns the dynamics of the parametric configuration. Although a comprehensive ablation analysis was conducted for the transition parameter (λ), resulting in an optimized system configuration, fundamental search parameters such as population size and maximum iteration count are still statically predefined within the algorithm. The absence of a dynamic control mechanism capable of autonomously updating these parameters according to image heterogeneity and pixel intensity distributions limits the flexibility and adaptability of the algorithm under varying contrast conditions. Finally, although quantitative metrics (PSNR, SSIM, and FSIM) together with statistical analyses (Pearson and Spearman correlations) mathematically demonstrate that the proposed algorithm preserves structural integrity, the study does not include an independent blinded subjective clinical validation performed by expert pathologists. The ultimate acceptance of the algorithm within medical device infrastructures and clinical decision-support systems will therefore require prospective validation studies supported by expert pathologist consensus and clinically interpretable evaluations.

## 7. Conclusions and Future Works

Accurate segmentation of cellular morphologies and tissue boundaries in histopathological images constitutes the foundation of computer-aided diagnostic systems in digital pathology. In this study, a novel hybrid metaheuristic framework, termed ARO-ALO, is proposed for the multi-level thresholding of heterogeneous tissue structures by synergistically combining the search characteristics of the ARO and ALO algorithms. Comprehensive experimental analyses demonstrate that the proposed algorithm not only achieves superior performance on the target colon adenocarcinoma dataset, but also exhibits a high degree of tissue-agnostic generalizability by outperforming contemporary SOTA metaheuristic models, such as RSA-SSA, on morphologically distinct OSCC and Pulmonary Circulation datasets. One of the most significant findings of the study is the objective identification of two fundamental structural weaknesses encountered by label-dependent deep learning models (e.g., CUNet3+) in histopathological image segmentation. First, the inherently discrete labeling structure of ground-truth masks employed in supervised learning leads to the loss of natural cellular intensity gradients and tissue texture details, thereby resulting in reduced structural similarity (SSIM). Second, deep learning models exhibit substantial overfitting to the limited illumination and staining characteristics of the training dataset, consequently producing high structural variance when evaluated on previously unseen test samples. In contrast to these limitations, the proposed unsupervised ARO-ALO algorithm does not require any labeled mask abstraction and instead directly identifies the natural intensity valleys within the original image histogram with high precision, thereby consistently preserving both structural integrity (SSIM) and diagnostic content (FSIM). The transition mechanism of the algorithm $\left(\lambda =0.6\right)$ establishes a logical balance between exploration and exploitation within the optimization space, effectively mitigating premature convergence and stochastic variance problems frequently encountered at high threshold levels (T ≥10) and maintaining them within clinically acceptable stability limits. This optimization stability, further supported through statistical correlation analyses, provides a strong methodological contribution to the literature by enabling the identification of cancerous regions without inducing morphological distortions. The obtained findings demonstrate that the proposed ARO-ALO algorithm possesses substantial potential as a robust, reference-mask-independent decision-support module that can be integrated into digital pathology workflows. In this context, future studies are planned to expand the clinical applicability and analytical scope of the proposed methodology along the following research directions:

- To overcome the computational bottlenecks of processing high-dimensional WSIs, future research will focus on integrating collaborative intelligence and dynamic split computing architectures [59,60]. Adapting these metaheuristic-driven multi-task learning frameworks will facilitate real-time, resource-efficient histopathological analysis across distributed GPU and edge environments.
- To further enhance the cellular analysis capability of the methodology, the framework will be extended to additional biomedical imaging modalities such as Differential Interference Contrast (DIC), Immunohistochemistry (IHC), and fluorescence microscopy, thereby enabling multi-modal segmentation capability.
- Next-generation supervised–unsupervised hybrid architectures will be designed by combining the rapid semantic localization capability of deep learning models (e.g., U-Net variants) with the fine-grained morphological thresholding capability of the proposed ARO-ALO algorithm in order to maximize diagnostic accuracy.
- In order to establish the proposed framework as a reliable clinical medical software system, future work will focus on validating the obtained segmentation results through prospective blinded evaluations based on expert pathologist consensus, together with the development of fully integrated end-to-end clinical decision-support platforms compatible with hospital information systems.
- To address the enormous storage requirements associated with high-resolution histopathological WSIs, clinically critical Regions of Interest (ROIs) isolated by the ARO-ALO algorithm are planned to be integrated into next-generation DNA-based data storage architectures. In this context, advanced metaheuristic optimization algorithms such as TC-HUR [61], which have demonstrated high data encoding capacity in the literature, will be utilized during the encoding of targeted medical data into synthetic DNA sequences, thereby establishing a multidisciplinary and long-term biological archiving infrastructure.

## Figures and Tables

**Table 2:** Parameter settings of the algorithms used.

Algorithm	Parameter
GWO	a: 2 → 0 (linear decreasing)
PSO	w: 0.4, $c_{1}$: 2.05, $c_{2}$: 2.05
ARO	α = 2
ALO	-
WDRIME	Woa Ratio = 0.4, Rime Ratio = 0.4, De Ratio = 0.2, wf = 0.8, cr = 0.9
ARO-ALO (Proposed)	λ = 0.6

**Table 3:** Specifications of used software versions, hardware.

Specification	Details
Operating System	Windows 10- 64 bit
Python Version	3.10
CPU	i5-1235U CPU 1.30GHz
RAM	16 GB 3200 MHz

**Table 4:** Summary statistics of PSNR for colon adenocarcinomas images according to algorithm and threshold level.

Algorithm	Threshold	PSNR	
		Mean	Standard Deviation
PSO	2	28.0920	0.4771
	4	27.7718	0.2158
	6	27.6857	0.2051
	8	27.6731	0.1949
	10	27.7612	0.3122
	12	27.7201	0.2449
GWO	2	28.3029	0.4854
	4	27.8760	0.2430
	6	27.7822	0.2024
	8	27.7695	0.2163
	10	27.9000	0.3179
	12	27.8940	0.2192
WDRIME	2	28.5558	0.4805
	4	28.1722	0.4334
	6	28.6520	0.5606
	8	28.3168	0.2713
	10	28.2830	0.5842
	12	28.4733	0.4692
ARO	2	27.9947	0.5031
	4	27.7450	0.2022
	6	27.6800	0.2053
	8	27.5993	0.1701
	10	27.6330	0.1864
	12	27.6366	0.2191
ALO	2	28.3033	0.4852
	4	27.8275	0.2371
	6	27.7853	0.2163
	8	27.7703	0.2430
	10	27.9003	0.2374
	12	27.8319	0.2220
ARO-ALO (Proposed)	2	28.0920	0.4771
	4	27.7737	0.2170
	6	27.6906	0.1935
	8	27.7018	0.2021
	10	27.7190	0.2452
	12	27.7427	0.2642

**Table 5:** Summary statistics of SSIM for colon adenocarcinomas images according to algorithm and threshold level.

Algorithm	Threshold	SSIM	
		Mean	Standard Deviation
PSO	2	0.4741	0.0761
	4	0.6260	0.0426
	6	0.7086	0.0522
	8	0.7549	0.0748
	10	0.7786	0.0766
	12	0.8067	0.0781
GWO	2	0.4656	0.0984
	4	0.6168	0.0547
	6	0.6825	0.0533
	8	0.7069	0.0745
	10	0.7561	0.0772
	12	0.7576	0.0592
WDRIME	2	0.2752	0.0863
	4	0.3800	0.1194
	6	0.4463	0.1286
	8	0.5166	0.1105
	10	0.5411	0.1320
	12	0.5458	0.1287
ARO	2	0.4991	0.0897
	4	0.6339	0.0518
	6	0.7085	0.0620
	8	0.7445	0.0815
	10	0.7876	0.0853
	12	0.7860	0.0932
ALO	2	0.4658	0.0987
	4	0.6139	0.0371
	6	0.6878	0.0523
	8	0.7199	0.0765
	10	0.7616	0.0819
	12	0.7766	0.0657
ARO-ALO (Proposed)	2	0.4741	0.0761
	4	0.6260	0.0426
	6	0.7155	0.0553
	8	0.7555	0.0801
	10	0.7889	0.0753
	12	0.8043	0.0821

**Table 6:** Summary statistics of FSIM for colon adenocarcinomas images according to algorithm and threshold level.

Algorithm	Threshold	FSIM	
		Mean	Standard Deviation
PSO	2	0.4979	0.0425
	4	0.6947	0.0661
	6	0.7786	0.0677
	8	0.8173	0.0644
	10	0.8376	0.0559
	12	0.8552	0.0574
GWO	2	0.4726	0.0304
	4	0.6582	0.0695
	6	0.7450	0.0618
	8	0.7631	0.0747
	10	0.8048	0.0561
	12	0.8128	0.0437
WDRIME	2	0.3577	0.0793
	4	0.4916	0.0889
	6	0.5576	0.0923
	8	0.6216	0.0745
	10	0.6540	0.0860
	12	0.6566	0.0826
ARO	2	0.5066	0.0495
	4	0.6994	0.0789
	6	0.7830	0.0745
	8	0.8211	0.0718
	10	0.8568	0.0562
	12	0.8564	0.0650
ALO	2	0.4727	0.0306
	4	0.6616	0.0623
	6	0.7461	0.0667
	8	0.7805	0.0592
	10	0.8194	0.0526
	12	0.8318	0.0407
ARO-ALO (Proposed)	2	0.4979	0.0425
	4	0.6941	0.0665
	6	0.7823	0.0701
	8	0.8192	0.0683
	10	0.8470	0.0607
	12	0.8595	0.0578

**Table 7:** Summary statistics of PSNR for benign tissue images according to algorithm and threshold level.

Algorithm	Threshold	PSNR	
		Mean	Standard Deviation
PSO	2	28.1031	0.4673
	4	27.8657	0.2544
	6	27.8623	0.3224
	8	27.8540	0.2722
	10	27.8654	0.3545
	12	27.8475	0.3616
GWO	2	28.2912	0.4355
	4	27.9696	0.2404
	6	27.9477	0.3004
	8	27.9748	0.1862
	10	27.9817	0.3490
	12	27.9935	0.2942
WDRIME	2	28.5635	0.3005
	4	28.5214	0.3066
	6	28.4450	0.4799
	8	28.0978	0.3803
	10	28.0299	0.2597
	12	27.9769	0.2484
ARO	2	28.2023	0.2899
	4	27.9324	0.1833
	6	27.9084	0.1852
	8	27.8100	0.1750
	10	27.9522	0.2017
	12	27.9689	0.2783
ALO	2	28.2851	0.4235
	4	27.9825	0.2415
	6	28.0284	0.2881
	8	27.9353	0.2692
	10	28.0834	0.2949
	12	27.9877	0.4159
ARO-ALO (Proposed)	2	28.1031	0.4673
	4	27.8703	0.2606
	6	27.8799	0.2846
	8	27.8429	0.2775
	10	27.8824	0.3391
	12	27.8734	0.3723

**Table 8:** Summary statistics of SSIM for benign tissue images according to algorithm and threshold level.

Algorithm	Threshold	SSIM	
		Mean	Standard Deviation
PSO	2	0.4431	0.0228
	4	0.6071	0.0320
	6	0.6964	0.0299
	8	0.7505	0.0291
	10	0.7910	0.0327
	12	0.8179	0.0339
GWO	2	0.4509	0.0117
	4	0.6079	0.0398
	6	0.6853	0.0357
	8	0.7214	0.0238
	10	0.7788	0.0508
	12	0.7905	0.0425
WDRIME	2	0.3193	0.0199
	4	0.4220	0.0351
	6	0.4940	0.0463
	8	0.5333	0.0839
	10	0.5891	0.0499
	12	0.5840	0.0532
ARO	2	0.4511	0.0114
	4	0.6072	0.0288
	6	0.6921	0.0222
	8	0.7382	0.0310
	10	0.7858	0.0115
	12	0.7983	0.0330
ALO	2	0.4516	0.0107
	4	0.6124	0.0298
	6	0.6898	0.0259
	8	0.7441	0.0312
	10	0.7740	0.0251
	12	0.8109	0.0269
ARO-ALO (Proposed)	2	0.4431	0.0228
	4	0.6075	0.0307
	6	0.6933	0.0306
	8	0.7470	0.0291
	10	0.7937	0.0370
	12	0.7998	0.0330

**Table 9:** Summary statistics of FSIM for benign tissue images according to algorithm and threshold level.

Algorithm	Threshold	FSIM	
		Mean	Standard Deviation
PSO	2	0.4616	0.0292
	4	0.6623	0.0282
	6	0.7599	0.0300
	8	0.8132	0.0211
	10	0.8432	0.0252
	12	0.8686	0.0243
GWO	2	0.4618	0.0307
	4	0.6668	0.0279
	6	0.7499	0.0331
	8	0.7927	0.0306
	10	0.8321	0.0463
	12	0.8446	0.0378
WDRIME	2	0.4057	0.0173
	4	0.5814	0.0314
	6	0.6617	0.0415
	8	0.7050	0.0601
	10	0.7471	0.0464
	12	0.7488	0.0248
ARO	2	0.4681	0.0270
	4	0.6618	0.0279
	6	0.7546	0.0285
	8	0.8024	0.0295
	10	0.8479	0.0142
	12	0.8561	0.0320
ALO	2	0.4620	0.0305
	4	0.6673	0.0269
	6	0.7577	0.0273
	8	0.8114	0.0306
	10	0.8423	0.0220
	12	0.8681	0.0225
ARO-ALO (Proposed)	2	0.4616	0.0292
	4	0.6615	0.0287
	6	0.7586	0.0295
	8	0.8099	0.0258
	10	0.8486	0.0276
	12	0.8561	0.0229

**Table 10:** General algorithm performance summary.

Algorithm	Class	PSNR		SSIM		FSIM	
		Mean	Std.	Mean	Std.	Mean	Std.
PSO	Adenocarcinoma	27.7599	0.2583	0.6955	0.064	0.7556	0.061
PSO	Benign Tissue	27.9010	0.3241	0.6897	0.030	0.7429	0.027
GWO	Adenocarcinoma	27.8882	0.2711	0.6715	0.065	0.7211	0.058
GWO	Benign Tissue	28.0103	0.2967	0.6779	0.033	0.7329	0.032
WDRIME	Adenocarcinoma	28.3458	0.4495	0.4491	0.118	0.5586	0.082
WDRIME	Benign Tissue	28.2861	0.3697	0.4951	0.048	0.6512	0.039
ARO	Adenocarcinoma	27.6812	0.2312	0.6975	0.077	0.7626	0.069
ARO	Benign Tissue	27.9535	0.2263	0.6843	0.027	0.7402	0.028
ALO	Adenocarcinoma	27.8772	0.2661	0.6757	0.068	0.7265	0.057
ALO	Benign Tissue	28.0380	0.3139	0.6870	0.025	0.7437	0.025
ARO-ALO	Adenocarcinoma	27.7549	0.2615	0.6986	0.064	0.7579	0.062
ARO-ALO	Benign Tissue	27.9049	0.3266	0.6876	0.029	0.7419	0.026

**Table 11:** A comparison of the generalizability and quantitative performance of the proposed ARO-ALO and SOTA meta-heuristic RSA-SSA algorithms across different histopathological datasets.

Dataset	Algorithm	Threshold	PSNR		SSIM		FSIM	
			Mean	Std.	Mean	Std.	Mean	Std.
Adenocarcinoma	RSA-SSA	2	28.0920	0.4771	0.4741	0.0761	0.4979	0.0425
		4	27.7759	0.2205	0.6272	0.0429	0.6956	0.0669
		6	27.7003	0.2146	0.7049	0.0565	0.7740	0.0682
		8	27.6306	0.2234	0.7680	0.0733	0.8273	0.0641
		10	27.7437	0.2519	0.7799	0.0925	0.8350	0.0703
		12	27.6995	0.2764	0.8021	0.0688	0.8557	0.0582
	ARO-ALO	2	28.0920	0.4771	0.4741	0.0761	0.4979	0.0425
		4	27.7737	0.2170	0.6260	0.0426	0.6941	0.0665
		6	27.6906	0.1935	0.7155	0.0553	0.7823	0.0701
		8	27.7018	0.2021	0.7555	0.0801	0.8192	0.0683
		10	27.7190	0.2452	0.7889	0.0753	0.8470	0.0607
		12	27.7427	0.2642	0.8043	0.0821	0.8595	0.0578
Benign Tissue	RSA-SSA	2	28.1031	0.4673	0.4431	0.0228	0.4616	0.0292
		4	27.8709	0.2610	0.6077	0.0305	0.6616	0.0286
		6	27.8274	0.3069	0.6944	0.0245	0.7580	0.0247
		8	27.8077	0.2724	0.7435	0.0439	0.8083	0.0290
		10	27.8810	0.3164	0.7816	0.0410	0.8373	0.0290
		12	27.9389	0.2899	0.7814	0.0404	0.8398	0.0331
	ARO-ALO	2	28.1031	0.4673	0.4431	0.0228	0.4616	0.0292
		4	27.8703	0.2606	0.6075	0.0307	0.6615	0.0287
		6	27.8799	0.2846	0.6933	0.0306	0.7586	0.0295
		8	27.8429	0.2775	0.7470	0.0291	0.8099	0.0258
		10	27.8824	0.3391	0.7937	0.0370	0.8486	0.0276
		12	27.8734	0.3723	0.7998	0.0330	0.8561	0.0229
Pulmonary Circulation Vessels Dataset	RSA-SSA	2	27.2420	0.2941	0.7285	0.0464	0.6153	0.0681
		4	27.9618	2.1171	0.7749	0.0805	0.7265	0.0869
		6	27.5041	0.5295	0.8070	0.1092	0.8026	0.0786
		8	28.0209	1.5796	0.7992	0.1223	0.8343	0.0702
		10	29.1674	2.0950	0.8538	0.0806	0.8843	0.0385
		12	28.7318	1.4453	0.8386	0.0661	0.8932	0.0237
	ARO-ALO	2	27.2420	0.2941	0.7285	0.0464	0.6153	0.0681
		4	28.0075	2.0973	0.7745	0.0804	0.7067	0.0730
		6	27.5025	0.5455	0.8005	0.1102	0.7983	0.0804
		8	27.8030	1.0142	0.7861	0.1179	0.8360	0.0646
		10	28.7421	1.7086	0.7989	0.1115	0.8504	0.0645
		12	28.8378	1.5028	0.8222	0.0825	0.8856	0.0515
OSCC	RSA-SSA	2	27.6648	0.2926	0.5679	0.0976	0.6163	0.0822
		4	27.8828	0.2733	0.7257	0.0815	0.7786	0.0679
		6	27.9024	0.2768	0.7973	0.0497	0.8409	0.0450
		8	27.9318	0.4504	0.8228	0.0583	0.8885	0.0385
		10	28.0604	0.4986	0.8447	0.0558	0.9010	0.0426
		12	27.9737	0.5709	0.8567	0.0309	0.9080	0.0417
	ARO-ALO	2	27.6648	0.2926	0.5679	0.0976	0.6163	0.0822
		4	27.8620	0.2741	0.7110	0.0605	0.7740	0.0617
		6	27.8691	0.3116	0.8004	0.0494	0.8427	0.0436
		8	27.9739	0.4694	0.8254	0.0413	0.8867	0.0333
		10	28.1284	0.5286	0.8463	0.0444	0.8999	0.0354
		12	28.0775	0.6463	0.8690	0.0463	0.9200	0.0336

**Table 12:** A Comparison of overall segmentation accuracy and stability between the fully supervised CUNet3+ deep learning model and the unsupervised ARO-ALO algorithm on the Pulmonary Circulation Vessels Dataset.

Algorithm	Statistic	PSNR	SSIM	FSIM	Dice	Jaccard	Hausdorff	BF-Score
CUNet3+	Mean	28.3436	0.5056	0.7350	0.8743	0.8085	33.7639	0.8666
	Std.	5.7667	0.0410	0.2316	0.1771	0.2252	67.4273	0.2526
ARO-ALO	Mean	27.8598	0.7865	0.7866	0.9534	0.9125	5.7445	0.9559
	Std.	0.9102	0.0896	0.0720	0.0696	0.0834	6.2400	0.0749

**Table 13:** Comparison of the average execution time per iteration for the evaluated algorithms.

Algorithm	Average Iteration Time (s)
GWO	0.0168
PSO	0.0170
WDRIME	0.0223
ARO	0.0282
ALO	0.0564
RSA-SSA	0.2295
ARO-ALO	0.0322

**Table 14:** Optimization accuracy, run-to-run stability, and computational performance of the evaluated algorithms under an equal FE budget (T=4 case study).

Algorithm	Stochastic Stability (Mean ± Std.)	PSNR (dB)	SSIM
Exhaustive Search (Ground Truth)	2502.7900	27.4617	0.6849
Hybrid ARO-ALO	2502.5875 ± 0.0256	27.4562	0.6864
CMA-ES	2502.7078 ± 0.0036	27.4617	0.6849
Standard Otsu DP	Deterministic	27.4617	0.6849
K-Means	Deterministic	27.3758	0.6935
Enlarged Budget ARO	2501.3352 ± 1.4627	27.5318	0.6933
ARO (Partial Budget)	2499.1405 ± 2.9070	27.4441	0.7048
ALO (Partial Budget)	2502.4644 ± 0.2116	27.4618	0.6826
Random Search	2456.2208 ± 86.2668	27.5514	0.5791

**Table 15:** Sensitivity analysis of the λ parameter.

λ	PSNR (dB)	SSIM	FSIM
0.4	27.2130	0.8743	0.9120
0.6	27.2758	0.8736	0.9135
0.8	27.3874	0.8687	0.9114

**Table 16:** Statistical ranking and significance analysis based on structural evaluation metrics across all benchmark datasets.

Algorithm	Average Rank (Friedman Test)	Wilcoxon Signed-Rank Test	Nemenyi Post-Hoc Test Result (α = 0.05)
ARO-ALO	1.85	–	Reference Method
PSO	2.30	0.384	No Significant Difference
RSA-SSA	2.85	0.082	No Significant Difference
ARO	3.40	0.041	Significant Difference
ALO	3.90	0.035	Significant Difference
GWO	4.80	0.006	Significant Difference
WDRIME	6.10	0.001	Significant Difference

## Data Availability

The original data presented in the study are openly available at [49,56,57].
